# Supplementary material for: Hydrogel-based lineage-specific gene therapy prevents recurrence of corticotroph tumors
Source: Mol Ther Oncol. 2026 Jun 2;34(3):201254. doi: 10.1016/j.omton.2026.201254 (PMC13292130; doi:10.1016/j.omton.2026.201254)
Supplement: Document S1. Figures S1–S8, Tables S1 and S2 [file mmc1.pdf]

OMTON, Volume *34*

Supplemental information

Hydrogel-based lineage-specific gene therapy prevents recurrence of corticotroph tumors

Junpeng Wang, Zezheng Fang, Jiahao Wang, Xu Han, Fan Feng, Runlu Zhang, Baoteng Han, Guangpan Sun, Yulin Zhang, and Shilei Ni

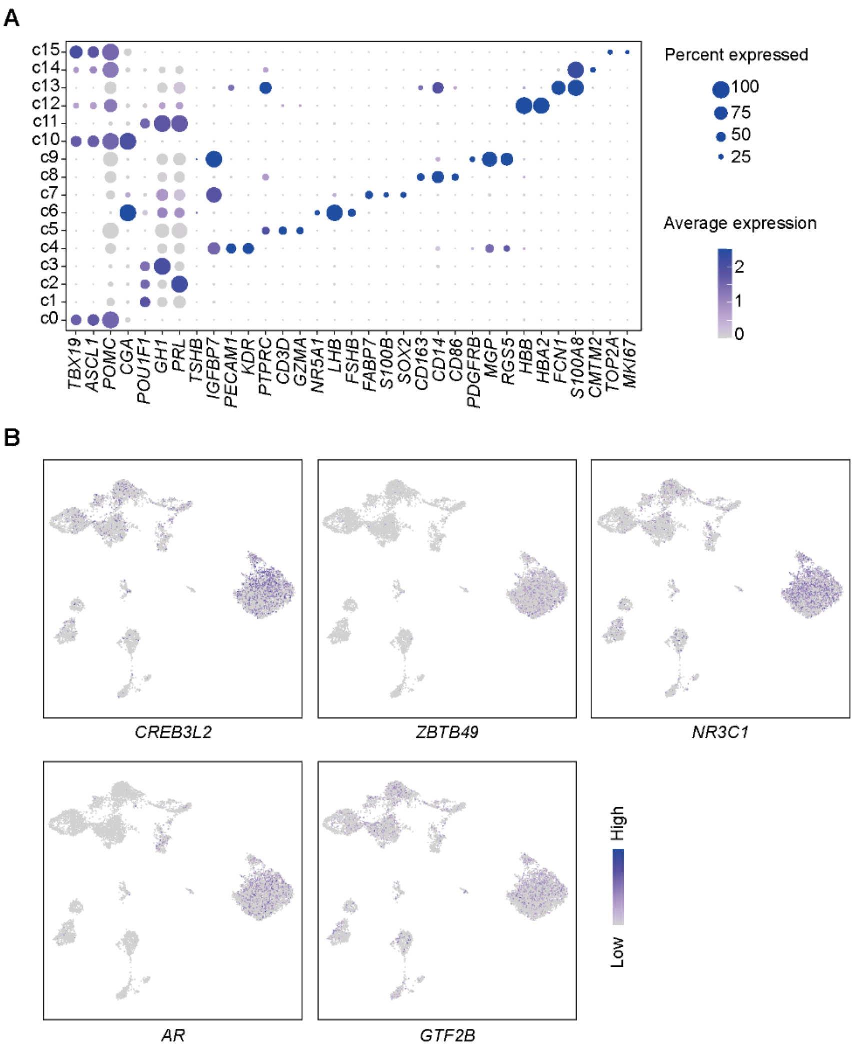

Figure S1. (A) Dot plot depicting marker gene expression across defined clusters. Dot color indicates the average expression level of the gene, and dot size represents the percentage of cells expressing the gene within each cluster. **(B)** UMAP visualization of specific transcription factors of TPIT-lineage cells. Color intensity (gray to blue) reflects increasing expression levels.

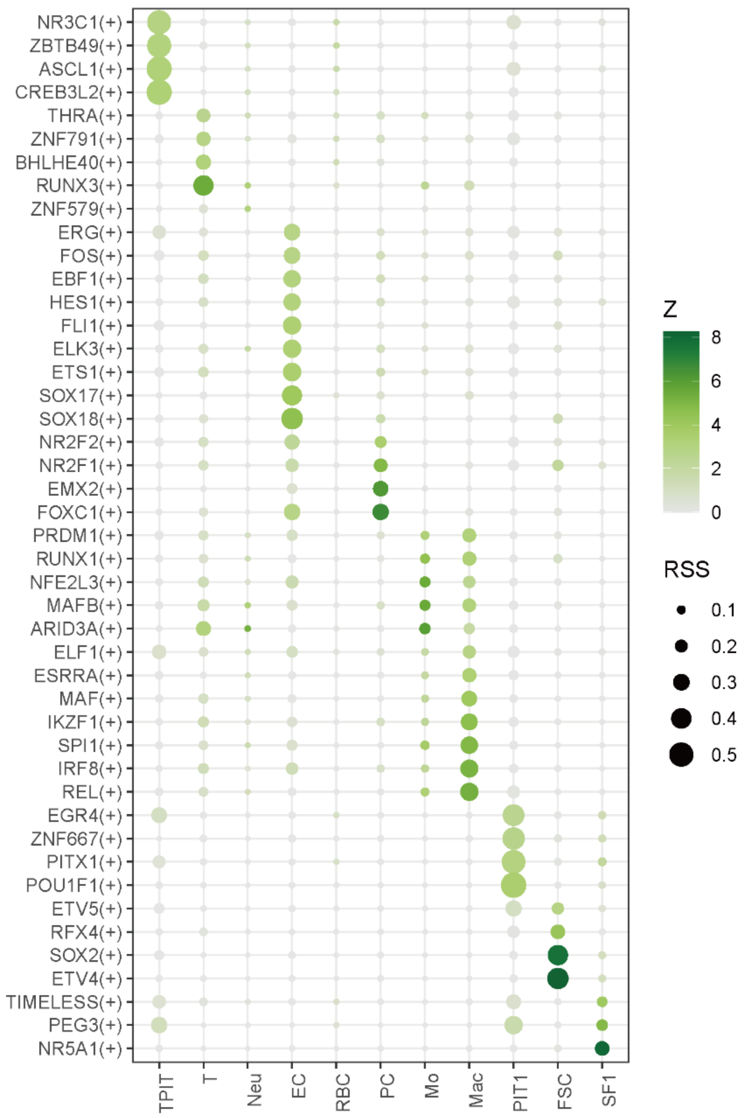

Figure S2. Dot plot showing activation status of cell-type-specific transcription factors (Z-score ≥ 2.5). Color represents Z-score (regulon activity), and dot size indicates RSS (regulon specificity) across clusters. TPIT, TPIT-lineage cells. T, T cells. Neu, neutrophil. EC, endothelial cell. RBC, red blood cells. PC, pericyte. Mo, monocyte. Mac, macrophage. PIT1, PIT1-lineage cells. FSC, folliculostellate cells. SF1, SF1-lineage cells.

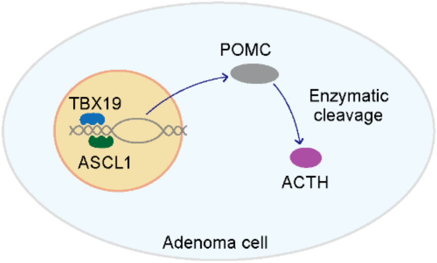

Figure S3. Schematic diagram of ACTH production process in corticotroph tumor cells.

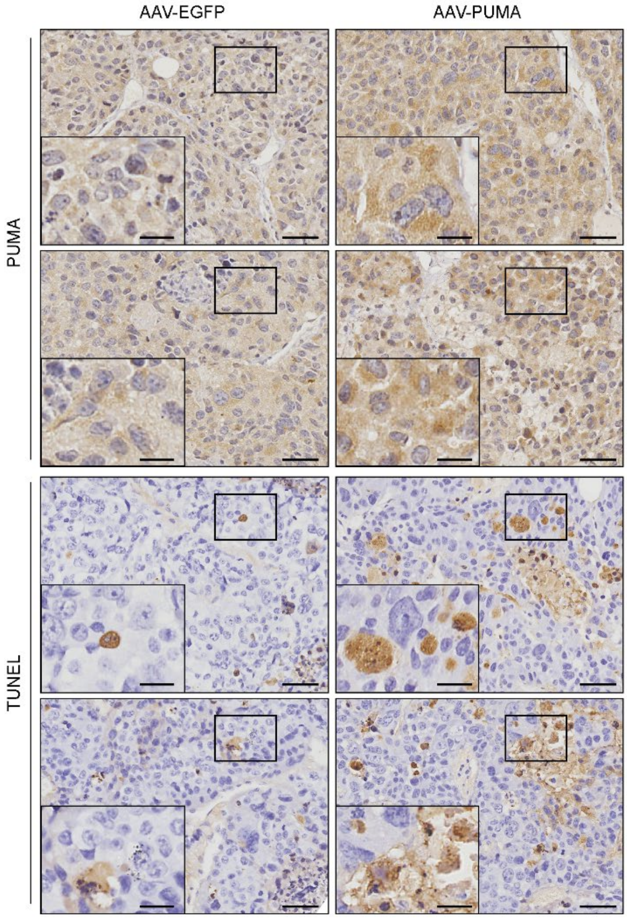

Figure S4. Representative IHC images of PUMA expression and TUNEL staining for apoptosis in tumor tissues. Scale bars: 50 µm (main panels), 20 µm (insets).

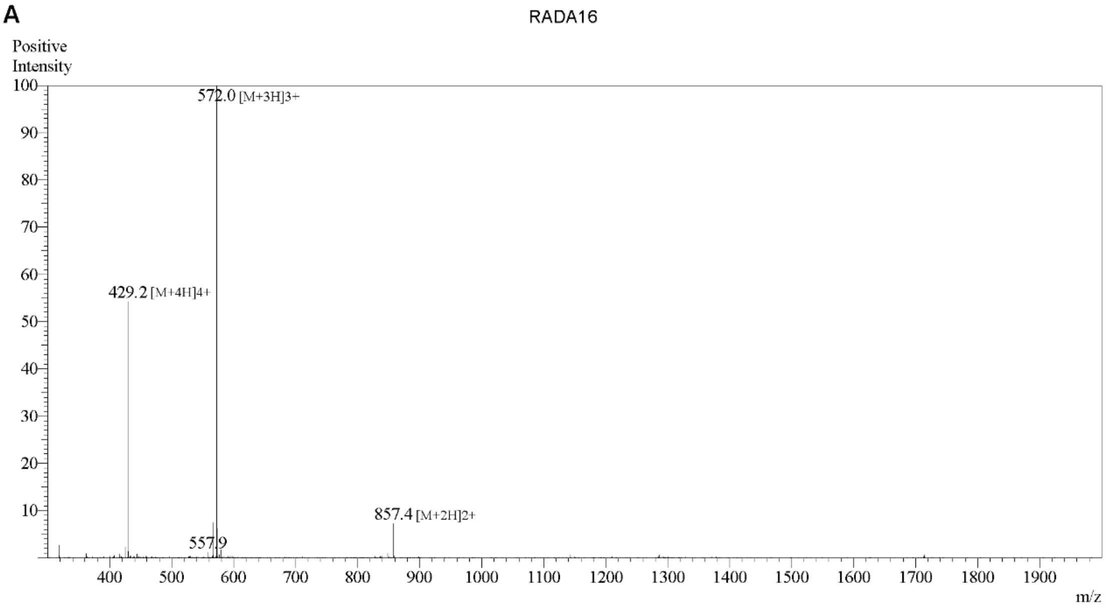

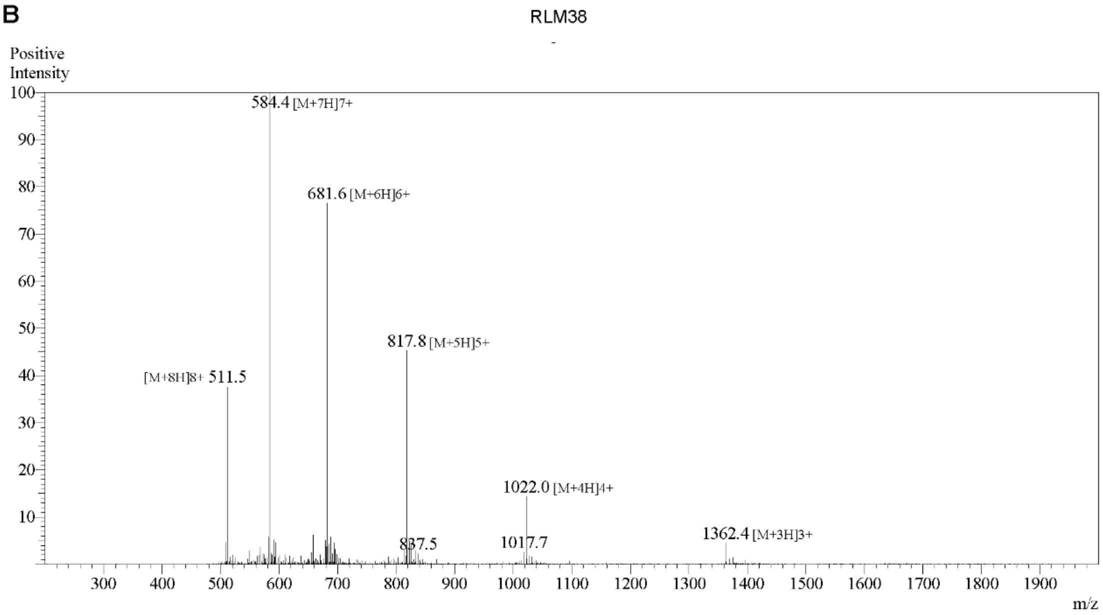

Figure S5. Mass spectrometry profiles of peptides RADA16 (A) and RLM38 (B).

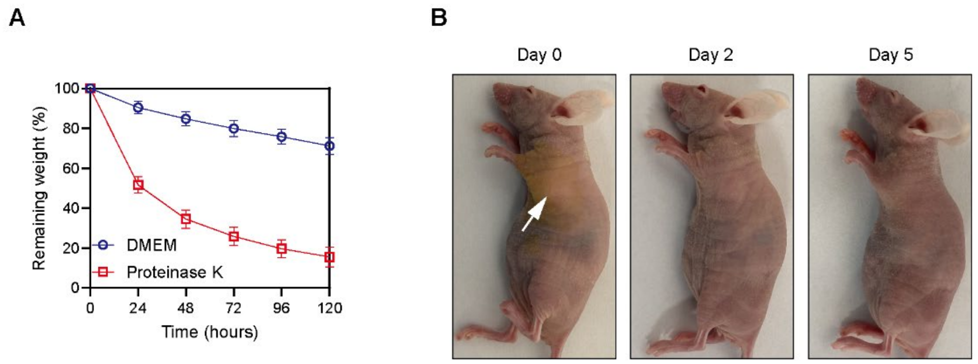

Figure S6. (A) Degradation of the blank hydrogel in DMEM with or without proteinase K. Data represent mean ± SD. n = 3. **(B)** Photograph of a mouse after subcutaneous injection of 30 µL blank hydrogel. The white arrow indicates the wheal formed at the injection site immediately following hydrogel administration.

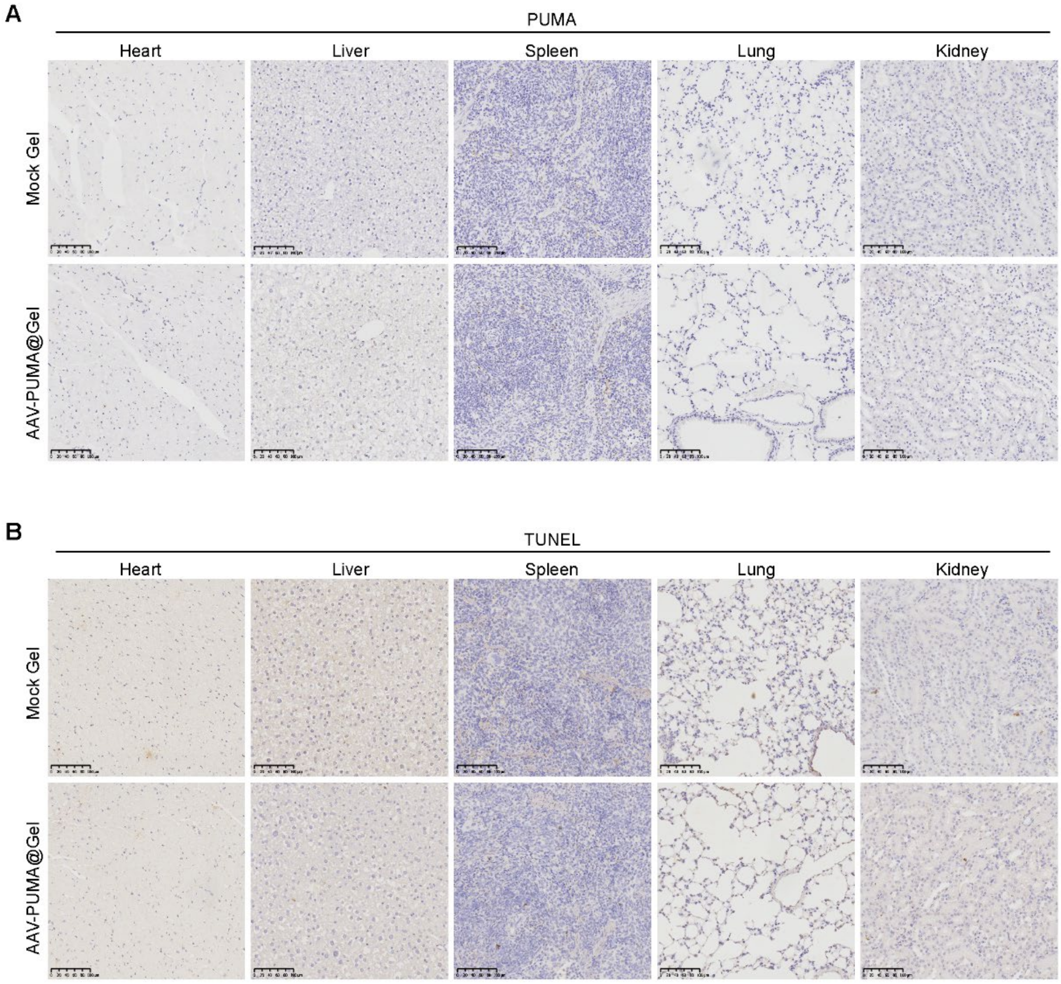

Figure S7. Representative IHC images of PUMA expression **(A)** and TUNEL staining for apoptosis **(B)** in heart, liver, spleen, lung, and kidney tissues. Scale bars: 100 µm.

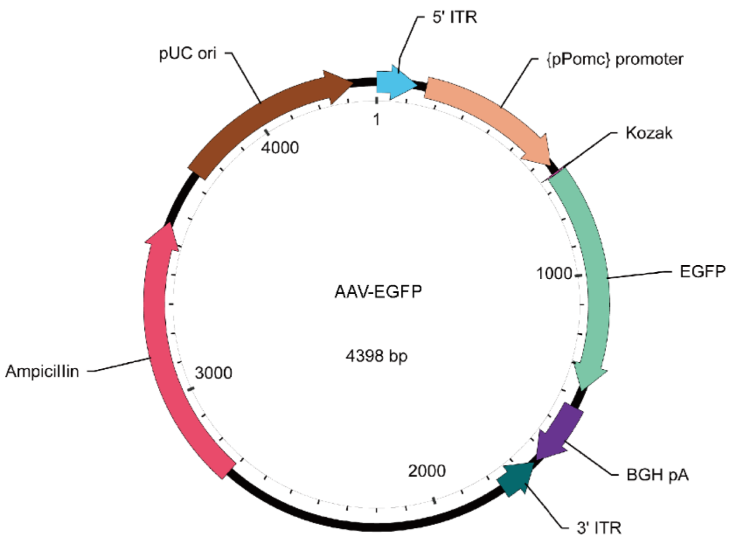

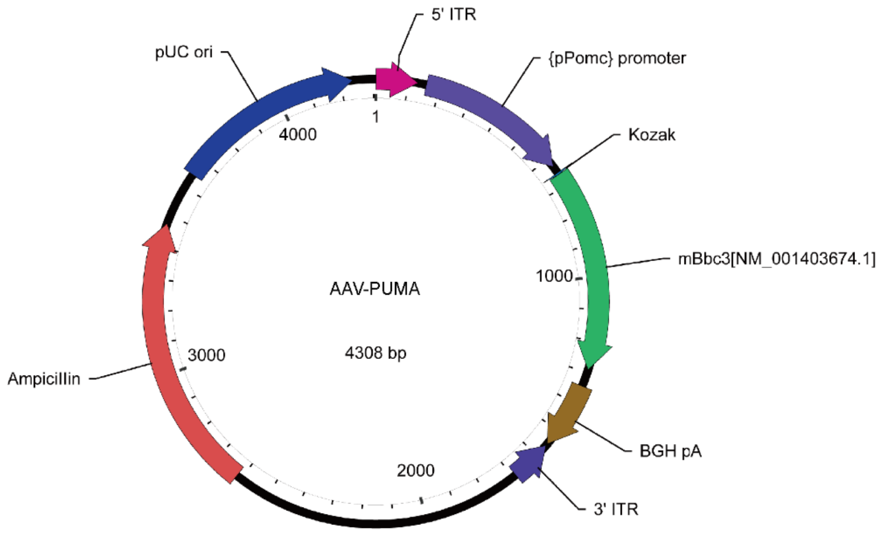

Figure S8. Plasmid maps of AAV-EGFP and AAV-PUMA.

Table S1. Sequences of siRNAs

siRNAs	Type	Sequences
siTBX19-1	sense	5'-GGAGAUAACAGCUCUCAAAdTdT-3'
	antisense	5'-UUUGAGAGCUGUUAUCUCCdTdT-3'
siTBX19-2	sense	5'-GGAUGUUCCCUGUCCUGAAdTdT-3'
	antisense	5'-UUCAGGACAGGGAACAUCCdTdT-3'
siNC	sense	5'-UUCUCCGAACGUGUCACGUdTdT-3'
	antisense	5'-ACGUGACACGUUCGGAGAAdTdT-3'

Table S2. Sequences of primers

Primers	Type	Sequences
Tbx19	forward	5'-GCCTGCTTAACGTGGTGGAA-3'
	reverse	5'-ACATCCGTCTGCCGTTCTTG-3'
Puma	forward	5'-CAGCACTTAGAGTCGCCCG-3'
	reverse	5'-GTGAGGGTCGGTGTCGATG-3'
Pomc	forward	5'-ATGCCGAGATTCTGCTACAGT-3'
	reverse	5'-CCACACATCTATGGAGGTCTGAA-3'
Gapdh	forward	5'-TGTCTCCTGCGACTTCAACA-3'
	reverse	5'- GGTGGTCCAGGGTTTCTTACT-3'
