# Supplementary material for: Hydrogel-based lineage-specific gene therapy prevents recurrence of corticotroph tumors
Source: Mol Ther Oncol. 2026 Jun 2;34(3):201254. doi: 10.1016/j.omton.2026.201254 (PMC13292130; doi:10.1016/j.omton.2026.201254)
Supplement: Document S2. Article plus supplemental information [file mmc2.pdf]

Molecular Therapy
Oncology
Original Article

Hydrogel-based lineage-specific gene therapy prevents recurrence of corticotroph tumors

Junpeng Wang,[1,2] Zezheng Fang,[1,2] Jiahao Wang,[1,2] Xu Han,[1,2] Fan Feng,[1,2] Runlu Zhang,[1,2] Baoteng Han,[1,2] Guangpan Sun,[1,2] Yulin Zhang,[1,2] and Shilei Ni[1,2]

[1]Department of Neurosurgery, Qilu Hospital, Cheeloo College of Medicine, Shandong University, 107 Wenhua Xi Road, Jinan 250012 Shandong, China; [2]Institute of Brain and Brain-Inspired Science, Shandong University, 107 Wenhua Xi Road, Jinan 250012 Shandong, China

Pituitary corticotroph tumors are the primary cause of Cushing's disease. Even after transsphenoidal surgery, residual tumor cells frequently persist, leading to a recurrence rate of approximately 20%. However, safe and effective adjuvant treatments for these residual lesions are lacking. In this study, we analyzed single-cell RNA sequencing data to characterize the transcriptional profile of corticotroph tumors. On the basis of the high activity of the *POMC* promoter and its transcriptional regulators, we designed an adeno-associated virus vector carrying the pro-apoptotic gene *Puma*, with expression controlled by the *Pomc* promoter for lineage-specific targeting. We also developed an injectable, self-assembling peptide hydrogel for the sustained local delivery of the *Puma* gene and tested its therapeutic efficacy in subcutaneous and post-resection mouse models. Results showed the *Pomc* promoter drove selective *Puma* expression in corticotroph tumor cells, inducing mitochondrial membrane potential loss and apoptosis *in vitro*. *In vivo*, the virus suppressed tumor growth and lessened systemic tumor effects; when delivered via the hydrogel into surgical cavities, it completely eliminated residual tumors without detectable toxicity in major organs. This targeted, localized strategy, thus has translational potential as an adjuvant therapy for reducing the recurrence of Cushing's disease.

INTRODUCTION

Cushing's disease is an endocrine disorder caused by the excessive secretion of adrenocorticotropic hormone (ACTH) from pituitary corticotroph tumors.[1,2] Long-term glucocorticoid excess leads to profound metabolic, cardiovascular, and immune complications.[1,2] Despite advances in transsphenoidal surgery, the recurrence or persistence of this disease remains a major clinical problem.[3] Large series and meta-analyses report that approximately one in five patients exhibits tumor regrowth after surgery, most often due to microscopic residual disease in anatomically challenging regions such as the cavernous sinus or suprasellar extension.[4]

For neurosurgeons, these residual lesions represent a persistent therapeutic gap. Current adjuvant options—including stereotactic radiosurgery, radiotherapy, and systemic medical therapy—can control the disease in some cases but often carry delayed efficacy, incomplete hormonal normalization, or risks to the surrounding neurovascular structures.[1,3] The neurosurgical field lacks precise, local, and durable therapy that directly targets residual corticotroph tumor cells without damaging the adjacent normal pituitary tissue or hypothalamic structures.[3,5]

The molecular profile of corticotroph tumors offers a promising basis for precision therapy.[6–9] A defining feature of these tumors is their expression of TBX19, a transcription factor restricted to the pituitary corticotroph lineage and known to directly activate the *POMC* promoter.[10–12] Our study revealed that high *POMC* transcriptional activity in corticotroph tumors is orchestrated by a regulatory network centered on *CREB3L2* and *ASCL1*. Given that proopiomelanocortin (POMC) is the precursor of ACTH,[11] this conserved transcriptional network drives excessive ACTH production and supports the secretory phenotype, which is consistently observed across tumors with diverse mutational backgrounds.[6]

Apoptosis induction represents a powerful way to eliminate tumor cells. p53 upregulated modulator of apoptosis (PUMA) is a BH3-only BCL-2 family protein that potently triggers mitochondrial outer membrane permeabilization and intrinsic apoptosis.[13] Prior studies in non-central nervous system (CNS) models have shown that the gene therapy delivery of PUMA can rapidly kill target cells.[14,15] However, no work has combined the potency of PUMA with the transcriptional selectivity of the *POMC* promoter to target pituitary tumors.

In this study, we designed an adeno-associated virus (AAV) vector in which the expression of the transgene is driven by the *Pomc* promoter. *In vitro* experiments demonstrated that transgene expression via this vector was highly specific to corticotroph tumor cells and was regulated by TBX19. The delivery of the *Puma* gene using

Received 17 October 2025; accepted 29 May 2026;
https://doi.org/10.1016/j.omton.2026.201254.

Correspondence: Yulin Zhang, Department of Neurosurgery, Qilu Hospital, Cheeloo College of Medicine, Shandong University, 107 Wenhua Xi Road, Jinan 250012 Shandong, China.
E-mail: yulin.zhang@sdu.edu.cn
Correspondence: Shilei Ni, Department of Neurosurgery, Qilu Hospital, Cheeloo College of Medicine, Shandong University, 107 Wenhua Xi Road, Jinan 250012 Shandong, China.
E-mail: nishilei@sdu.edu.cn

 © 2026 The Authors.
Published by Elsevier Inc. on behalf of The American Society of Gene and Cell Therapy.
This is an open access article under the CC BY license (http://creativecommons.org/licenses/by/4.0/).

this AAV system effectively induced apoptosis in corticotroph tumor cells and suppressed tumor growth in mouse models. To address the clinical challenge of residual tumor tissue following surgery, we further developed a peptide-based hydrogel system for the sustained intratumoral (i.t.) delivery of the AAV vector. A single administration of the AAV-loaded hydrogel into the postoperative tumor cavity achieved complete tumor eradication in preclinical models, offering a promising strategy for preventing recurrence after surgical resection.

RESULTS

High *POMC* expression is autonomously maintained in corticotroph tumors

To understand how high levels of ACTH are synthesized in corticotroph tumors without triggering cellular stress responses, we performed bioinformatic analyses on their single-cell RNA sequencing (scRNA-seq) data. After the identification of highly variable genes and the application of dimensionality reduction, 11,309 cells from the tumor and the adjacent normal gland were clustered into 16 distinct populations, including TPIT (T-box transcription factor TBX19) lineage cells (*TBX19*$^+$), PIT1 (pituitary-specific positive transcription factor 1) lineage cells (*POU1F1*$^+$), SF1 (steroidogenic factor 1) lineage cells (*NR5A1*$^+$), folliculostellate cells (FSC, *S100B*$^+$*SOX2*$^+$),[10,16] myeloid cells, and vascular wall cells (Figures 1A and S1A).

PIT1 lineage cells, defined by the expression of the transcription factor *POU1F1*, typically include somatotrophs (*GH1*$^+$), lactotrophs (*PRL*$^+$), and thyrotrophs (*TSHB*$^+$); whereas SF1 lineage cells, defined by the expression of the transcription factor *NR5A1*, include gonadotrophs (*FSHB*$^+$/*LHB*$^+$).[10] Single-cell analysis revealed GH1 and PRL coexpression as well as negativity for both hormones within the PIT1 lineage, likely representing transitional or silent cell states (Figures 1A and S1A). Notably, from this dataset, thyrotrophs (*TSHB*$^+$) were not identified, which may be attributed to their scarcity in pituitary tissue.[10] Corticotroph tumor cells were predominantly TPIT lineage cells (Figure 1B), which exhibited high expression of *POMC* as well as the previously identified lineage-defining transcription factors *TBX19* and *ASCL1* (Figure 1C).[9,12]

We further applied single-cell regulatory network inference and clustering (SCENIC)[17] analysis to infer the key transcription factors for each cell type (Figure S2). The analysis identified the top six transcription factors with regulatory activity in corticotroph tumor cells (Figures 1D and S1B). Among them, CREB3L2 has been implicated in translational efficiency and secretory capacity in pituitary cells,[8] while ASCL1 is a critical regulator of corticotroph differentiation and maturation, capable of directly binding to the *POMC* promoter to drive its expression.[9] Additionally, protein export and oxidative phosphorylation pathways were significantly activated in corticotroph tumors (Figure 1E), reflecting their increased energy demands and capacity for hormone secretion. Excessive POMC is proteolytically processed into ACTH,[18] ultimately leading to Cushing's disease (Figure S3).

Although TBX19 was not ranked among the top regulators in the SCENIC analysis, functional evidence confirms its role in *Pomc* expression in mouse corticotroph tumor cells. Similar to ASCL1, TBX19 binds directly to the *Pomc* promoter (Figure 1F). Small interfering RNA (siRNA)-mediated silencing of *Tbx19* in the AtT20 corticotroph tumor cell line significantly reduced *Pomc* transcript levels and lowered ACTH secretion (Figures 1G–1J). These findings demonstrate that the high transcriptional and translational output of *Pomc* in corticotroph tumors is sustained by a coordinated network of transcription factors. This mechanistic insight provides a compelling rationale for developing targeted therapeutic strategies that exploit this lineage-specific regulatory architecture.

Pomc promoter drives PUMA overexpression to selectively eliminate corticotroph tumor cells

Given the high transcriptional activity of *Pomc* and the elevated protein synthesis characteristic of corticotroph tumors, we developed an AAV vector in which the transgene expression is driven by the *Pomc* promoter (Figure 2A). To evaluate the cell-type specificity of this vector, we transduced the mouse cell lines AtT20 (corticotroph tumor), GL261 (glioma), and RAW264.7 (macrophage-like) with AAV-EGFP. Because GL261 and RAW264.7 cells do not express the pituitary-specific transcription factor TBX19 (Figure 2B), *Pomc* promoter activity is expected to be restricted. Consistent with this, EGFP expression was only observed in AtT20 cells, with no detectable expression in GL261 or RAW264.7 cells at 48 h post-transduction (Figure 2C). Furthermore, AAV-EGFP did not affect cell viability at this time point. In contrast, the AAV-mediated overexpression of the pro-apoptotic protein PUMA (AAV-PUMA) significantly reduced AtT20 cell viability at 48 h post-transduction (Figure 2D). Pre-knockdown of TBX19 reduced PUMA overexpression compared to AAV-PUMA treatment alone (Figure 2E). Flow cytometry analysis confirmed that AAV-PUMA significantly induced apoptosis and mitochondrial membrane potential loss in AtT20 cells. Notably, the pre-knockdown of TBX19 partially reversed the cytotoxic effects of AAV-PUMA (Figures 2F–2I). These results indicated that PUMA overexpression driven by the *Pomc* promoter exerts specific cytotoxicity against corticotroph tumor cells.

AAV-mediated PUMA overexpression inhibits corticotroph tumor progression *in vivo*

To evaluate the *in vivo* anti-tumor efficacy of AAV-PUMA, we established a subcutaneous (s.c.) corticotroph tumor mouse model and randomized mice into two groups: AAV-EGFP (control) and AAV-PUMA (treatment). When tumors reached a volume of approximately 25 mm^3, mice received i.t. injections of the respective AAV vectors (Figure 3A). With tumors' progression, both groups exhibited progressive weight loss, skin thinning, and limb atrophy, suggesting that the corticotroph tumors induced systemic endocrine and metabolic dysfunction. AAV-PUMA-treated mice showed significantly less weight loss compared to the control group (mean [SD], 15.99 [0.72] g vs. 15.01 [0.62] g, $p = 0.023$) (Figure 3B). Furthermore, because excessive ACTH secretion by corticotroph tumors is the underlying cause of Cushing's disease, the observed reduction in serum

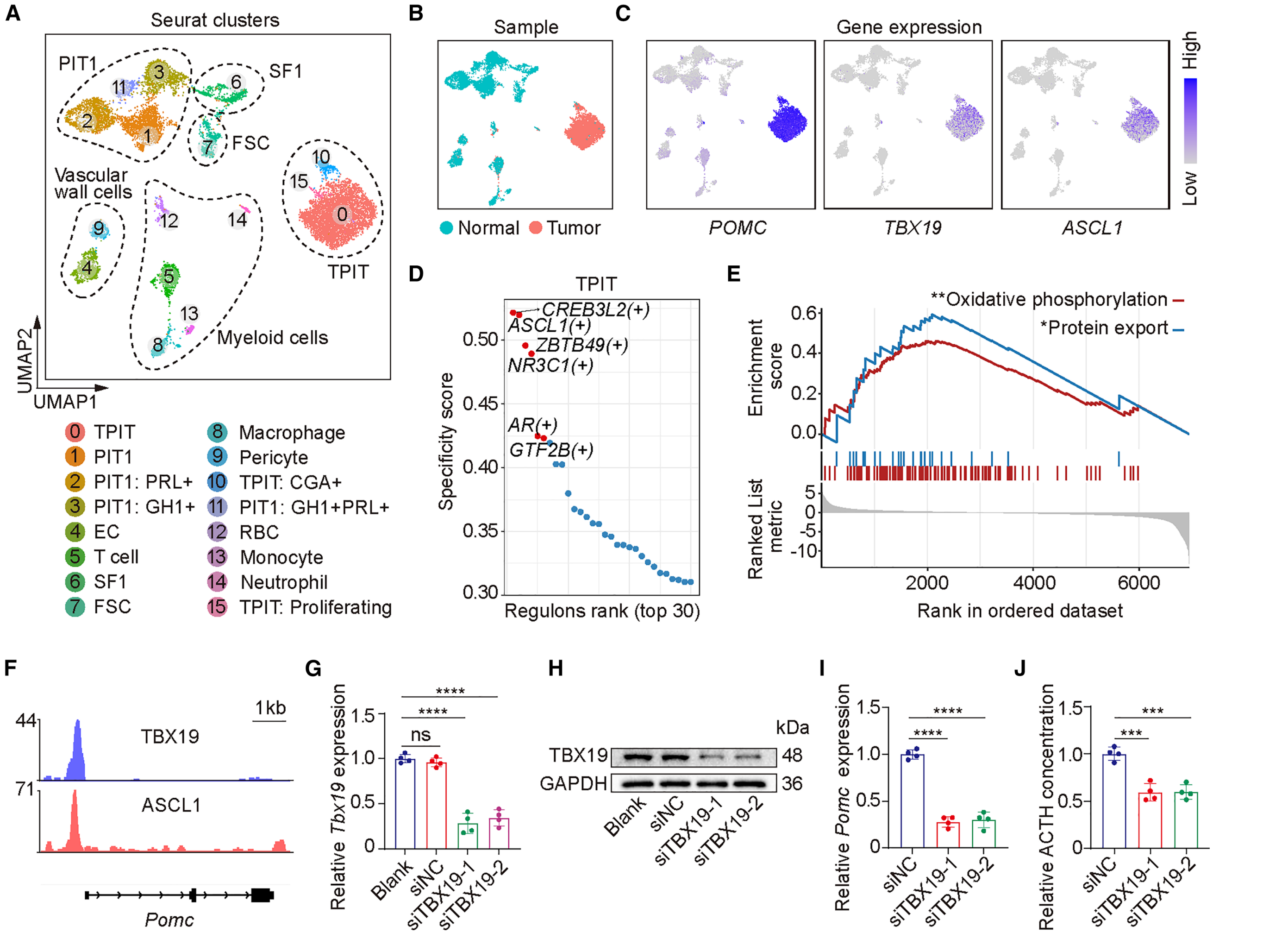

Figure 1. Lineage-specific transcription factors mediate high *POMC* expression in corticotroph tumors

(A) UMAP plot showing the annotated cell types identified in corticotroph tumor samples. EC, endothelial cell; FSC, folliculostellate cells; RBC, red blood cells. (B) UMAP plot highlighting the tumor cells (in red) and the adjacent normal gland cells (in green). (C) UMAP visualization of known TPIT-lineage cell markers. Color intensity (gray to blue) reflects increasing expression levels. (D) Scatterplot showing specificity scores of the top 30 regulators in TPIT-lineage cell clusters. The top six regulators are highlighted in red and labeled. (E) GSEA shows significant enrichment of the protein export (adjusted $p = 0.037$, BH) and oxidative phosphorylation (adjusted $p = 0.003$, BH) pathways in TPIT-lineage cell clusters. (F) Integrative genomics viewer browser screenshot showing TBX19 and ASCL1 binding at the *Pomc* promoter region. (G) Relative mRNA expression of *Tbx19* in AtT20 cells after knockdown. $n = 4$. (H) Western blot analysis showing TBX19 protein levels in AtT20 cells after knockdown. (I) Reduced *Pomc* mRNA expression in AtT20 cells after *Tbx19* knockdown. $n = 4$. (J) Decreased ACTH levels in AtT20 cells after *Tbx19* knockdown. siTBX19-1 vs. siNC, $p = 0.0004$; siTBX19-2 vs. siNC, $p = 0.0003$. $n = 4$. Statistical comparisons were performed using two-tailed Student's *t* tests. Data represent mean ± SD (G, I, and J). *$p < 0.05$, **$p < 0.01$, ***$p < 0.001$, ****$p < 0.0001$. ns, not significant.

ACTH levels following AAV-PUMA treatment reflects a direct therapeutic benefit (mean [SD], 41.4 [7.96] pg/mL vs. 66.2 [11.21] pg/mL, $p = 0.0038$) (Figure 3C). Tumor growth was also significantly suppressed, as evidenced by the longitudinal tumor volume measurements (day 19, mean [SD], 60.42 [24.59] mm^3 vs. 172.7 [28.42] mm^3, $p < 0.0001$) (Figures 3D and 3E). Immunohistochemical analysis revealed increased PUMA expression and enhanced apoptosis in tumors treated with AAV-PUMA (Figures 3F and S4). These results demonstrate that the AAV-mediated delivery of PUMA into corticotroph tumors effectively inhibits tumor progression *in vivo* and ameliorates tumor-associated endocrine and metabolic disturbances, highlighting its potential as a targeted therapeutic strategy.

Self-assembling peptide hydrogels enable the sustained i.t. delivery of AAV

To model a combined therapeutic strategy involving pro-apoptotic gene delivery and surgical intervention for corticotroph tumors, we developed an injectable, self-assembling peptide hydrogel designed for implantation into the tumor resection cavity. The hydrogel is composed of RADA16 and RLM38 peptides (peptide mass

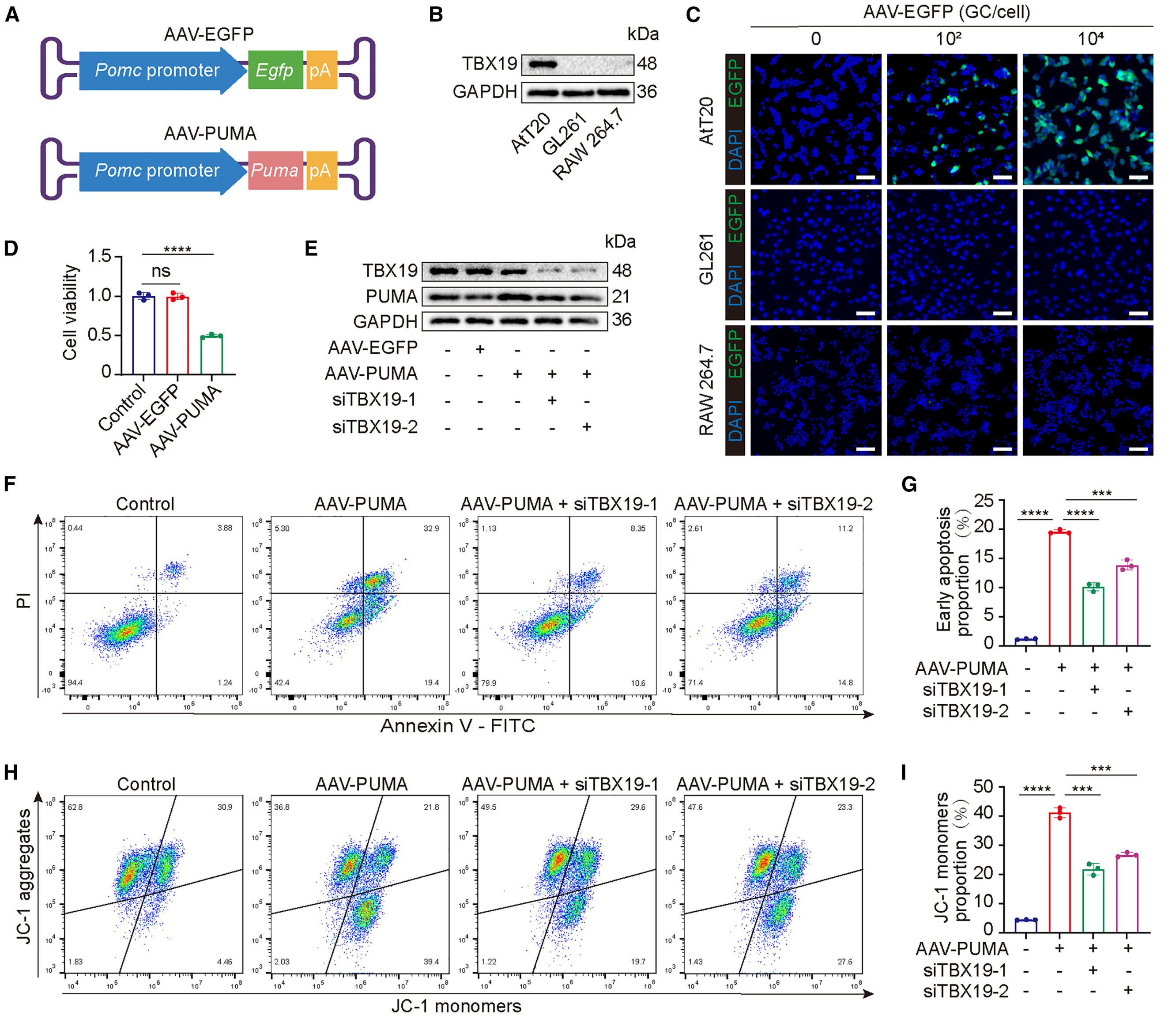

Figure 2. *Pomc* promoter-driven PUMA overexpression induces apoptosis in corticotroph tumor cells

(A) Schematic illustration of the recombinant adeno-associated virus (AAV) vector containing the *Pomc* promoter. (B) Western blot analysis showing the expression of TBX19 in various cell lines. (C) EGFP expression in cell lines infected with AAV-EGFP at titers of 0, 10^2, and 10^4 GC/cell for 48 h. Nuclei were stained with DAPI. GC, gene copy. Scale bars, 50 μm. (D) Relative cell viability of AtT20 cells infected with AAV-EGFP or AAV-PUMA at a titer of 10^4 GC/cell for 48 h $n = 3$. (E) Western blot analysis showing the effect of *Tbx19* knockdown on PUMA expression in AtT20 cells after AAV infection. (F) Representative flow cytometry analysis showing the effect of *Tbx19* knockdown on apoptosis in AtT20 cells after AAV-PUMA infection. (G) Quantification of early apoptosis in AtT20 cells following AAV-PUMA infection after *Tbx19* knockdown. AAV-PUMA + siTBX19-2 vs. AAV-PUMA, $p = 0.0004$. $n = 3$. (H) Representative flow cytometry analysis showing the effect of *Tbx19* knockdown on mitochondrial membrane potential (ΔΨm) in AtT20 cells after AAV-PUMA infection. (I) Quantification of JC-1 monomers in AtT20 cells following AAV-PUMA infection after *Tbx19* knockdown. JC-1, 5,5′,6,6′-tetrachloro-1,1′,3,3′-tetraethyl-imidacarbocyanine iodide. AAV-PUMA + siTBX19-1 vs. AAV-PUMA, $p = 0.0002$; AAV-PUMA + siTBX19-2 vs. AAV-PUMA, $p = 0.0002$. $n = 3$. Statistical comparisons were performed using two-tailed Student's t tests. Data represent mean ± SD (D, G, and I). *$p < 0.05$, **$p < 0.01$, ***$p < 0.001$, ****$p < 0.0001$. ns, not significant.

spectra shown in Figure S5), where RLM38 is a fusion peptide comprising a myelin basic protein-derived sequence linked to RADA16 (Figure 4A).[19–21] When mixed in aqueous solution at a mass ratio of 9:1, the RADA16 motifs undergo self-assembly into β-sheet nanostructures (Figure 4B). Adjusting the pH to neutral triggers the rapid formation of a transparent, mechanically supportive hydrogel (Figure 4C). Scanning electron microscopy (SEM) revealed a well-defined, porous three-dimensional network (Figure 4D). Rheological analysis demonstrated typical hydrogel behavior: the storage modulus (G') exceeded the loss modulus (G'') across the

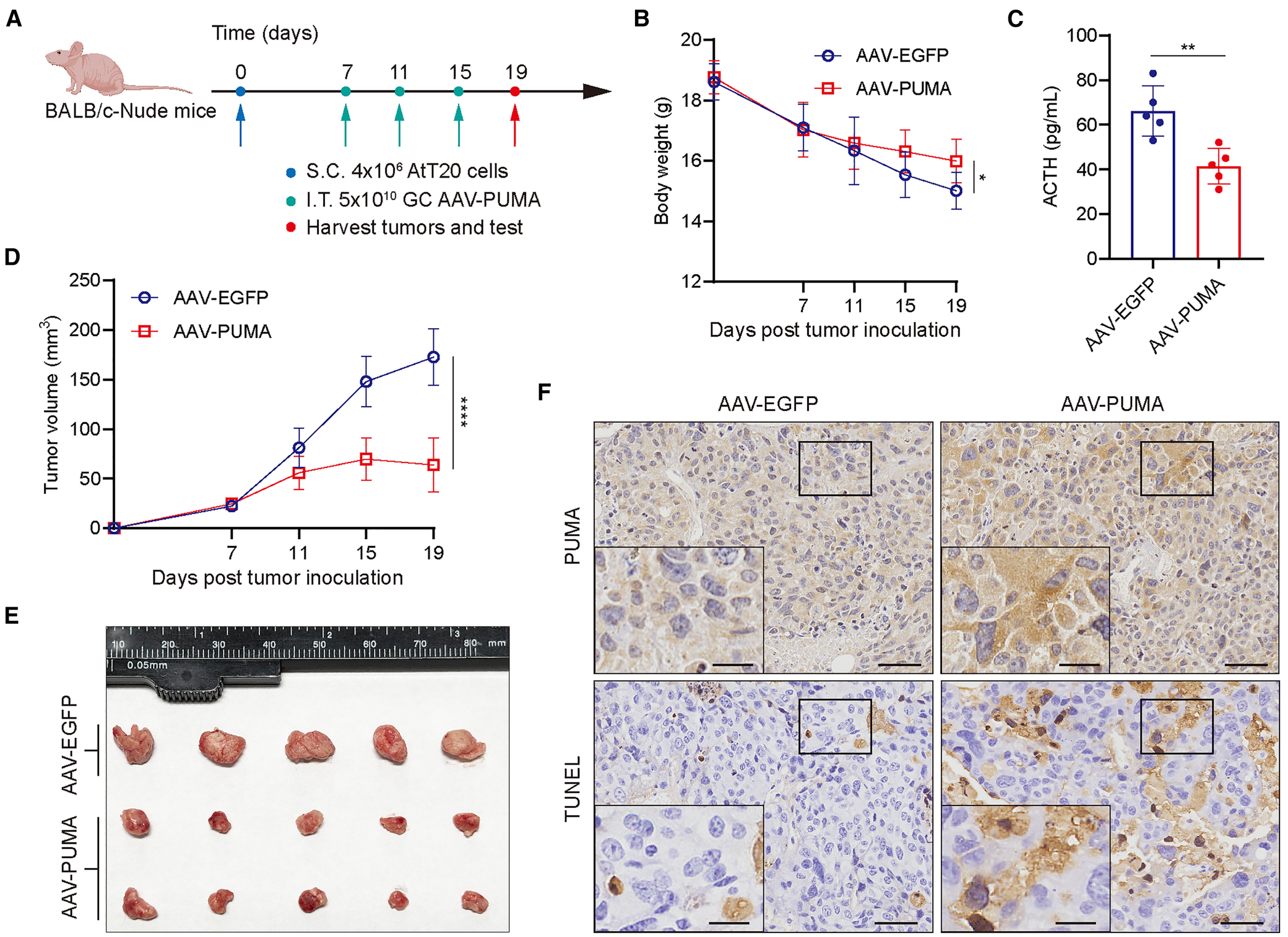

Figure 3. AAV-mediated PUMA overexpression inhibits corticotroph tumor progression *in vivo*

(A) Schematic illustration of the treatment regimen in AtT20 tumor-bearing mice. s.c., subcutaneous; i.t., intratumoral. (B) Body weight change curves for mice after indicated treatment. Day 19, AAV-PUMA vs. AAV-EGFP: 15.99 ± 0.72 g vs. 15.01 ± 0.62 g, $p = 0.023$. $n = 5$ (AAV-EGFP group) and $n = 10$ (AAV-PUMA group) mice. (C) Serum ACTH levels in mice 19 days post-tumor inoculation. AAV-PUMA vs. AAV-EGFP: 41.4 ± 7.96 pg/mL vs. 66.2 ± 11.21 pg/mL, $p = 0.0038$. $n = 5$ mice/group. (D) Tumor volume change curves for mice after indicated treatment. Day 19, AAV-PUMA vs. AAV-EGFP: 60.42 ± 24.59 mm^3 vs. 172.7 ± 28.42 mm^3, $p < 0.0001$. $n = 5$ (AAV-EGFP group) and $n = 10$ (AAV-PUMA group). (E) Gross morphology of excised subcutaneous tumors. (F) Representative IHC images of PUMA expression and TUNEL staining for apoptosis in tumor tissues. Scale bars, 50 μm (main images), 20 μm (insets). Statistical comparisons were performed using two-tailed Student's *t* tests. Data represent mean ± SD (B, C, and D). *$p < 0.05$, **$p < 0.01$, ***$p < 0.001$, ****$p < 0.0001$. ns, not significant.

tested frequency range, confirming the formation of a solid-like gel (Figures 4E and 4F). Additionally, the hydrogel exhibited pronounced shear-thinning properties, with viscosity sharply decreasing as shear rate increased from 0.1 to 100 s^{-1}, indicating excellent injectability and self-healing capacity (Figure 4G).

In vitro degradation assays using proteinase K showed near-linear mass loss over 120 h, with approximately 84.4% degradation (Figure S6A). *In vivo*, s.c. injection of the hydrogel into the dorsal region of nude mice resulted in gradual and complete resorption within several days, with no signs of skin damage or inflammation (Figure S6B). To assess its utility for sustained viral vector delivery, we examined AAV release kinetics from the hydrogel in Dulbecco's modified Eagle's medium (DMEM), with or without proteinase K. In the presence of proteinase K, AAV release was accelerated, reaching ~80.6% by day 5 (Figure 4H). Notably, i.t. injection of AAV-EGFP-loaded hydrogels into s.c. corticotroph tumors led to progressive increases in EGFP expression in tumor tissue over time, as monitored by continuous fluorescence imaging (Figure 4I). Overall, these results demonstrate that the self-assembling peptide hydrogel is a promising platform for adjuvant gene therapy following surgical resection.

AAV-PUMA-loaded hydrogel eradicates residual tumors after intracavitary delivery

To test the therapeutic efficacy of AAV-PUMA delivered via peptide hydrogel (AAV-PUMA@Gel) in the postsurgical setting, we established an s.c. corticotroph tumor resection model (Figure 5A).

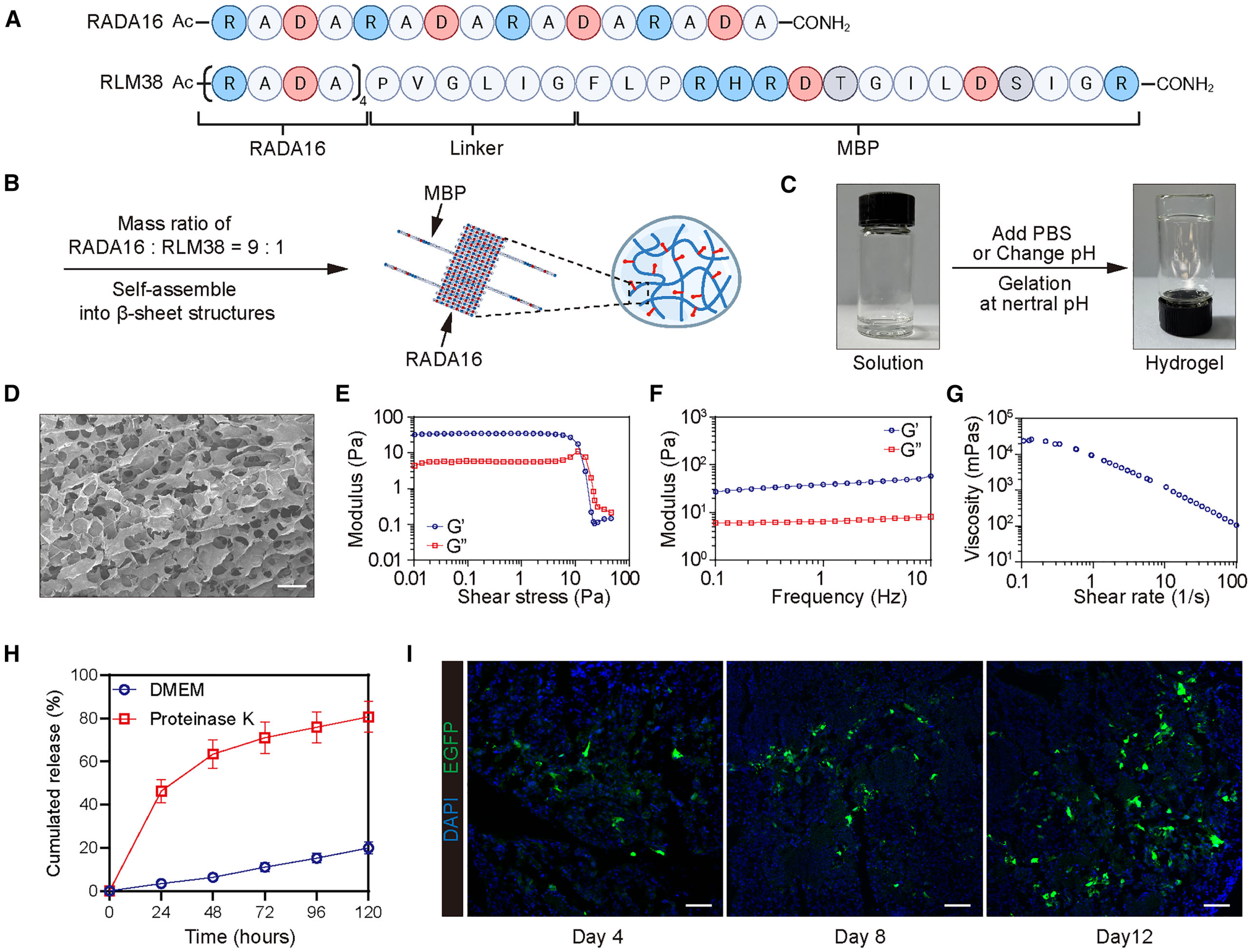

Figure 4. Self-assembled peptide hydrogel enables sustained intratumoral delivery of AAV

(A) Schematic illustration of the peptide sequences RADA16 and RLM38. (B) Illustration of the self-assembly process and formation of the peptide-based hydrogel. (C) Photographs of peptide gelator solutions and the hydrogel. (D) SEM images of the hydrogel. Scale bars, 100 μm. (E) Rheological analysis showing the storage modulus (G') and loss modulus (G'') under increasing shear stress, indicating the hydrogel's mechanical stability. (F) Angular frequency dependence of G' and G'' in the range of 0.1–10 rad/s, demonstrating viscoelastic properties. (G) Shear viscosity as a function of shear rate, confirming the hydrogel's pseudoplastic (shear-thinning) behavior. (H) *In vitro* release kinetics of AAV from the hydrogel in the presence or absence of proteinase K. $n = 3$. (I) *In vivo* expression of EGFP in corticotroph tumors of mice at 4, 8, and 12 days post-treatment. Scale bars, 100 μm.

Twelve days after the implantation of luciferase-expressing AtT20 cells (AtT20-Luc), visible tumors were surgically removed (Figure 5B), and the resection cavity was immediately filled with either AAV-PUMA@Gel or the mock gel.

Bioluminescence imaging confirmed a sharp decline in tumor signal immediately after resection, from $\sim 10^9$ to 10^6–10^7 photons/s/cm^2/sr, reflecting successful removal of the primary mass (Figures 5C and 5D). By day 28, the AAV-PUMA@Gel group exhibited markedly lower recurrence than the control group (median radiance: 1.96×10^4 vs. 9.21×10^8, $p = 0.0286$). Correspondingly, serum ACTH levels were significantly reduced in treated animals (mean [SD], 21.25 [3.3] pg/mL vs. 54.25 [4.57] pg/mL, $p < 0.0001$), indicating improved endocrine function (Figure 5E). Immunohistochemistry (IHC) confirmed no evidence of off-target expression or abnormal apoptosis in the major organs (Figure S7), supporting a favorable safety profile.

In summary, the intracavitary delivery of AAV-PUMA via a self-assembling hydrogel effectively suppressed residual tumors, prevented recurrence, and restored hormonal balance in this preclinical model. These findings highlight the potential of hydrogel-based, promoter-driven gene therapy as a precise, neurosurgeon-compatible adjuvant strategy for corticotroph tumors.

DISCUSSION

Residual or recurrent corticotroph tumors represent one of the most frustrating realities in the surgical treatment of Cushing's disease.

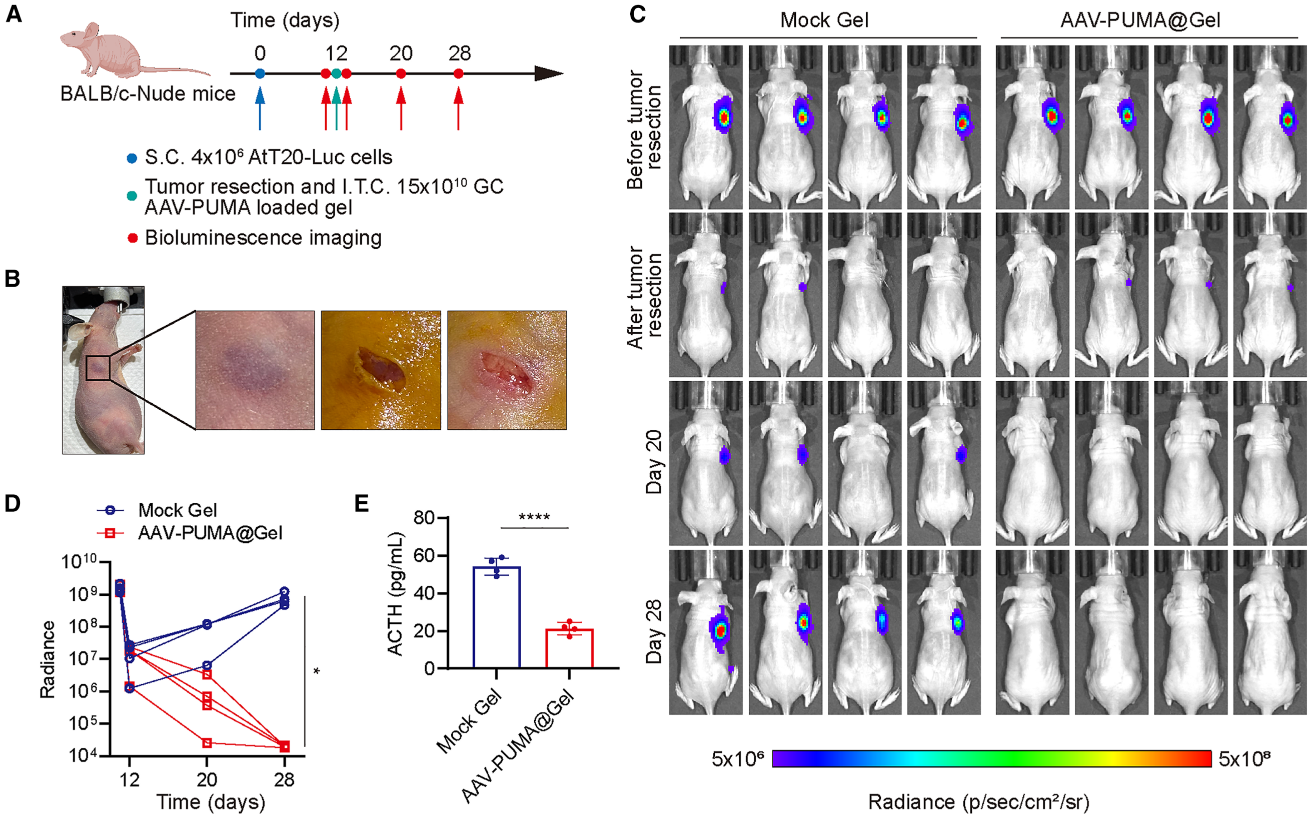

Figure 5. AAV-PUMA-loaded hydrogel prevents postoperative relapse of corticotroph tumors after intracavity delivery

(A) Schematic illustration of the experimental design. (B) Tumor debulking surgery in AtT20-Luc tumor-bearing mice on day 12 after tumor cell inoculation. i.t.c., intratumoral cavity. (C) *In vivo* bioluminescence imaging. Radiance: photons/sec/cm^2/sr. (D) Bioluminescence quantification. AAV-PUMA@Gel vs. Mock Gel, median radiance: 1.96×10^4 vs. 9.21×10^8, $p = 0.0286$. $n = 4$ mice/group. (E) Serum ACTH levels in mice 28 days post-tumor inoculation. AAV-PUMA@Gel vs. Mock Gel, 21.25 ± 3.3 pg/mL vs. 54.25 ± 4.57 pg/mL, $p < 0.0001$. $n = 4$ mice/group. Statistical comparisons were performed using Mann-Whitney test (D) and two-tailed Student's *t* test (E). Data represent mean ± SD (E). *$p < 0.05$, **$p < 0.01$, ***$p < 0.001$, ****$p < 0.0001$. ns, not significant.

Even in expert hands, tumor cells infiltrating the cavernous sinus, clinging to the dura, or scattered at the surgical margins can escape removal.[22] These surviving cells are not inert—they retain the capacity to reignite hypercortisolism, erode patient quality of life, and undermine the perceived "success" of surgery. Despite decades of incremental advances, we still lack a therapy designed to act in the critical postoperative window, when the tumor bed is exposed and the biological stakes are highest.[23–26]

The concept behind this work is deliberately different from conventional adjuvant therapy.[1] We did not seek to add another systemic drug or deliver a generalized local toxin. Instead, we designed a system that recognizes corticotroph tumor cells by their most defining molecular trait: a transcriptional identity centered on *POMC*. This lineage-locked promoter is not just a marker—it is a switch we can wire to an apoptotic payload. By coupling it to PUMA, a mitochondrial death signal,[13] we turned the tumor's own identity into its point of vulnerability. In doing so, we created a therapy that is silent in surrounding tissue yet lethal in its intended target.

Delivery is not an afterthought here—it is the other half of the innovation. The injectable, self-assembling peptide hydrogel was chosen because it aligns with neurosurgical reality: it can be placed in the resection cavity within minutes, conform to irregular spaces, and release its payload gradually over days.[27,28] In our preclinical models, this meant that tumor cells left behind after simulated surgery were exposed continuously to the targeted vector until eradicated. The result was not just tumor suppression but complete absence of regrowth in the treated group, accompanied by normalization of ACTH levels and no evidence of collateral tissue damage.

These findings also carry mechanistic weight. Our study confirms that corticotroph tumors are transcriptionally organized to sustain high *POMC* expression, with TBX19, ASCL1, and CREB3L2 orchestrating a program that meets the metabolic and secretory demands of chronic hormone overproduction. By inserting PUMA into this same transcriptional circuit, we created a strategy that co-opts the tumor's metabolic investment for its own destruction. This represents more than targeted therapy—it is identity-driven therapy, in which lineage commitment itself is the therapeutic gatekeeper.

In the broader neuro-oncology context, this study contributes to a shift from tissue-agnostic local therapies to promoter- and state-specific interventions. Intracavitary delivery is not new—carmustine wafers,[29] viral oncolytics,[30] and other local agents have been trialed in brain tumors—but these approaches typically rely on nonspecific toxicity.[31] Our approach brings selectivity into the equation, enabling a level of precision that could be transformative in anatomically constrained and functionally critical regions such as the sella.

We recognize the translational steps ahead. Orthotopic pituitary models are needed to test the interplay between vector distribution, cerebrospinal fluid dynamics, and hydrogel degradation in the unique environment of the sellar region. Immune responses to the vector must be mapped, particularly in the context of repeat exposure or pre-existing AAV immunity. Scalability of dosing from mouse to human volumes requires careful modeling, and intraoperative workflow integration must be tested in real surgical settings.

The potential scope of this platform extends beyond corticotroph tumors. By swapping the *POMC* promoter for other lineage-specific regulatory elements, the same hydrogel-vector architecture could be applied to other pituitary tumors, hypothalamic tumors, or even non-pituitary CNS tumors with stable promoter signatures. Combining promoter-targeted gene therapy with other modalities—such as stereotactic radiosurgery for margin control or immune checkpoint blockade to prevent recurrence—could create layered, durable local control strategies.

Ultimately, what we propose is a rethinking of how we approach residual CNS tumors: to treat them not as a distant concern for later intervention, but as an immediate, molecularly identifiable target at the time of surgery. In Cushing's disease, this philosophy could shift the goal from "maximal safe resection" to "maximal safe eradication"—combining surgical skill with molecular precision in a single operation. If successful, this shift would not just improve outcomes in Cushing's disease but could redefine the standard for adjuvant therapy across neuro-oncology.

MATERIALS AND METHODS

scRNA-seq data analysis

Processed scRNA-seq data were obtained from the GEO dataset GSE208108.[32] Data analysis was performed using the Seurat package (v.4.5.0).[33] To filter out low-quality cells, those failing to meet the following criteria were excluded: (1) detecting more than 200 genes; (2) detecting more than 500 UMIs (unique molecular identifiers); (3) mitochondrial UMI count accounting for less than 25% of the total. After sctransform-based normalization and principal component analysis, the Harmony package was used to remove batch effects. K-nearest neighbor and Louvain algorithms were employed to construct the intercellular distance matrix and perform cell clustering, respectively. Uniform manifold approximation and projection (UMAP) was used for nonlinear dimensionality reduction. Differentially expressed genes were identified using the "FindMarkers" function in the Seurat package. Pathway enrichment comparisons between different cell cluster combinations were analyzed using the gene set enrichment analysis (GSEA) method.

Transcription factor analysis

The SCENIC python workflow (v.0.12.1) was used to identify active transcription factors in the single-cell expression matrix and construct gene regulatory networks.[17] The human transcription factor gene list, ranked data of genes and motifs in the 10 kb region upstream and downstream of transcription start sites, and motif-to-transcription factor mapping data were obtained from https://resources.aertslab.org/cistarget/. The AUCell algorithm was used to calculate the activity score of each regulator in individual cells, evaluating the activation status of regulators at the single-cell level. The function "calcRSS" in the SCENIC package was used to calculate the relative specificity of regulators in different cell types, quantifying the enrichment of regulator activity in specific cell types. Chromatin immunoprecipitation sequencing (ChIP-seq) data were obtained from the GEO dataset GSE64483.[9] The integrative genomics viewer software (v.2.19.1) was used to visualize the binding peaks of ASCL1 and TBX19 to the *Pomc* gene.

Cell culture and activity assay

The AtT20 cell line (AtT-20/D16v-F2) was purchased from the American Type Culture Collection (Manassas, VA, USA). GL261 and RAW264.7 cells were obtained from the Chinese Academy of Sciences Cell Bank. These cells were cultured in DMEM (Gibco, USA) supplemented with 10% fetal bovine serum (Gibco, USA), 100 μg mL^{-1} streptomycin sulfate, and penicillin G sodium at 37°C. All cell lines tested negative for mycoplasma contamination. After 48 h of treatment, CCK-8 reagent (10 μL/well) (NCM Biotech, Suzhou, China) was added to AtT20 cells cultured in 96-well plates, and the absorbance at 450 nm (OD450) was measured using the EnSpire Multimode Plate Reader (PerkinElmer, Waltham, MA, USA).

Synthesis and transfection of siRNA and viral vectors

siRNAs targeting the mouse *Tbx19* gene (siTBX19) and a negative control siRNA (siNC) were synthesized by Obio (Shanghai, China). The sequences of all siRNAs are provided in Table S1. Hieff Trans Universal Transfection Reagent for siRNA transfection was purchased from Yeasen (Shanghai, China). Construction of AAV-EGFP and AAV-PUMA plasmids, viral packaging, and purification were performed by VectorBuilder (Guangzhou, China). Plasmid maps are provided in Figure S8. Lentiviruses expressing luciferase were obtained from Obio (Shanghai, China). AtT20 cells were transfected with siRNA or viruses according to the manufacturer's instructions.

Quantitative RT-PCR

Total RNA extraction and reverse transcription were performed using the RNA-quick purification kit (ES Science, Shanghai, China) and HiScript III RT SuperMix (Vazyme, Nanjing, China), respectively. AAV genomic DNA was extracted using a genomic DNA mini extraction kit (Beyotime, Shanghai, China). Primers were synthesized by BioSune (Shanghai, China). The sequences of all primers

are provided in Table S2. Quantitative reverse-transcription PCR (RT-qPCR) was performed using a LightCycler 480 II Real-Time PCR system (Roche, Basel, Switzerland) and Hieff qPCR SYBR Green Master Mix (Yeasen, Shanghai, China).

Western blot assay

Total cellular protein was extracted using RIPA (radioimmunoprecipitation assay) lysis buffer, and protein concentration was quantified using a BCA kit (Beyotime, Shanghai, China). Total protein was separated by SDS-PAGE (Vazyme, Nanjing, China) and transferred to a polyvinylidene fluoride (PVDF) membrane (Pall, USA). The membrane was incubated with primary antibodies overnight at 4°C, followed by a 1 h incubation with secondary antibodies (#RS0002, 1:10,000, Immunoway) at room temperature. Immunoreactive bands were visualized using an Enhanced Chemiluminescent Substrate (NCM Biotech, Suzhou, China) and imaged with a ChemiDoc XRS+ Imaging System (Bio-Rad, Hercules, CA, USA). The following primary antibodies were used: rabbit anti-TBX19 (#CAB10481, 1:500, Assay Genie), rabbit anti-PUMA (#55120-1-AP, 1:500, Proteintech), and rabbit anti-GAPDH (#YM8394, 1:2,000, Immunoway).

Flow cytometry and fluorescence imaging

An Annexin V-FITC/PI apoptosis detection kit (Yeasen, Shanghai, China) and an enhanced mitochondrial membrane potential assay kit with JC-1 (Beyotime, Shanghai, China) were used to detect early apoptosis and mitochondrial membrane potential loss, respectively. At low mitochondrial membrane potential, the JC-1 probe fails to accumulate in the mitochondrial matrix and exists as monomers emitting green fluorescence. After 48 h of treatment, AtT20 cells were trypsinized into single-cell suspensions. Cell counts were performed using the NovoCyte D3000 Flow Cytometer (Agilent Technologies, Santa Clara, CA, USA). For observing EGFP expression in cells or tumor tissue sections, nuclei were stained with 4',6-diamidino-2-phenylindole (DAPI, Beyotime, Shanghai, China). Images were captured using a fluorescence microscope (Leica Camera AG).

Synthesis and characterization of hydrogels

RADA16 and RLM38 peptides were custom-synthesized by GenScript (Nanjing, China) using solid-phase synthesis. RADA16 and RLM38 were mixed in an aqueous solution at a mass ratio of 9:1 and sonicated for 10 min to a final concentration of 1% (10 mg/mL, w/v). All peptide solutions were filtered through a 0.22 μm filter for sterilization. These self-assembling peptide solutions were converted into hydrogels by adding PBS buffer, cell culture medium, or adjusting the pH to neutral. The microstructure of hydrogels was examined using SEM. Rheological analysis was performed using a rheometer (Anton-Paar MCR302). For preparing the AAV-hydrogel sustained-release system, pre-quantified AAV was mixed with the initial aqueous solution. Shear-thinning kinetics of hydrogels were characterized by linear shear rate scanning from 0.1/s to 100/s. In *in vitro* degradation experiments, hydrogels were incubated in DMEM with or without proteinase K (5 U/mL) at 37°C for 5 days, and residual weight was measured at intervals. The release curve was plotted by detecting AAV genomes in the supernatant after gel degradation using PCR.

Animal studies

Animal experiments were approved by the Institutional Animal Care and Use Committee of Qilu Hospital, Shandong University (approval number: DWLL-202500092). To establish corticotroph tumor models, 100 μL DMEM suspensions containing 4×10^6 AtT20 or AtT20-Luc cells were injected into the right dorsal s.c. space of 7-week-old BALB/c-nude mice. Tumor volume was calculated as (length $\times$ width^2)/2. *In vivo* tumor growth was monitored by bioluminescence imaging using the IVIS Spectrum system (PerkinElmer; MA, USA). When s.c. tumors reached a volume of 25 mm^3, 5 μL of AAV solution containing 5×10^{10} gene copies (GC) was injected intratumorally every 4 days for a total of 3 injections. To simulate residual tumors after surgery, when tumor radiance reached 10^9, visible tumors were surgically resected, and 30 μL of AAV-loaded hydrogel sustained-release system (15×10^{10} GC) was injected into the surgical cavity. To evaluate gene expression after AAV delivery via peptide hydrogels, 5 μL of peptide hydrogel containing 5×10^{10} GC AAV-EGFP was injected intratumorally when s.c. tumors reached 50 mm^3, and frozen sections were prepared at scheduled time points.

ELISA

A Mouse ACTH enzyme-linked immunosorbent assay (ELISA Kit) was purchased from Elabscience (#E-EL-M0079, Wuhan, China). ACTH concentrations in cell supernatants or mouse serum were measured according to the manufacturer's instructions. Absorbance at 450 nm (OD450) was measured using the EnSpire Multimode Plate Reader (PerkinElmer, Waltham, MA, USA).

IHC

Immunohistochemical staining was performed on paraffin sections of mouse s.c. corticotroph tumors. The IHC detection kit (including secondary antibody and DAB [3,3'-diaminobenzidine] chromogen) was purchased from Immunoway (#RS0050). The primary antibody against PUMA was purchased from Proteintech (#55120-1-AP, 1:500). A colorimetric TUNEL apoptosis assay kit (#C1098, Beyotime, Shanghai, China) was used to detect apoptosis in sections. Immunohistochemical staining was performed according to the manufacturer's instructions.

Statistical analyses

Data are presented as means and standard deviations (SD). Differences between two independent groups were analyzed using two-tailed Student's *t* test or Mann-Whitney test. All statistical data were derived from biological replicates. A *p* value <0.05 was considered statistically significant. GraphPad Prism (v.10, GraphPad Software, CA, USA) was used for statistical analysis.

DATA AND CODE AVAILABILITY

The data and unique biological materials that support the findings of this study are available from the corresponding author (nishilei@sdu.edu.cn), upon reasonable request.

ACKNOWLEDGMENTS

This work was supported by the National Natural Science Foundation of China (82111530202, 82172740, and 82303810), the Natural Science Foundation of Shandong Province (ZR2022ZD17, ZR2021LSW008, and ZR2023QH224), the China Postdoctoral Science Foundation (2022M721967 and 2024T170524), the Innovation Project of Jinan Science and Technology Bureau (2021GXRC065), the Taishan Scholar Foundation of Shandong Province (no. tstp20230656 and no. tsqnz20221165), and the research project of Jinan Microecological Biomedicine Shandong Laboratory (JNL-2023004C, JNL-2023013D).

AUTHOR CONTRIBUTIONS

S.N., Y.Z., and J.W. conceived and designed the experiments; J.W. performed most of the experiments; J.W. and Z.F. contributed to the data analysis; J.W., X.H., F.F., R.Z., G.S., and B.H. assisted with the experiments and data analysis; J.W. participated in the discussion of data analysis, interpretation, and presentation, and drafted the manuscript; S.N. and Y.Z. reviewed and edited the manuscript. All authors contributed to the drafting of the manuscript and the critical review of its important intellectual content.

DECLARATION OF INTERESTS

The authors declare no competing interests.

SUPPLEMENTAL INFORMATION

Supplemental information can be found online at https://doi.org/10.1016/j.omton.2026.201254.

REFERENCES

1. Fleseriu, M., Auchus, R., Bancos, I., Ben-Shlomo, A., Bertherat, J., Biermasz, N.R., Boguszewski, C.L., Bronstein, M.D., Buchfelder, M., Carmichael, J.D., et al. (2021). Consensus on diagnosis and management of Cushing's disease: a guideline update. Lancet Diabetes Endocrinol. *9*, 847–875. https://doi.org/10.1016/S2213-8587(21)00235-7.

2. Nieman, L.K., Castinetti, F., Newell-Price, J., Valassi, E., Drouin, J., Takahashi, Y., and Lacroix, A. (2025). Cushing syndrome. Nat. Rev. Dis. Primers *11*, 4. https://doi.org/10.1038/s41572-024-00588-w.

3. Fleseriu, M., Varlamov, E.V., Hinojosa-Amaya, J.M., Langlois, F., and Melmed, S. (2023). An individualized approach to the management of Cushing disease. Nat. Rev. Endocrinol. *19*, 581–599. https://doi.org/10.1038/s41574-023-00868-7.

4. Stroud, A., Dhaliwal, P., Alvarado, R., Winder, M.J., Jonker, B.P., Grayson, J.W., Hamizan, A., Harvey, R.J., and McCormack, A. (2020). Outcomes of pituitary surgery for Cushing's disease: a systematic review and meta-analysis. Pituitary *23*, 595–609. https://doi.org/10.1007/s11102-020-01066-8.

5. Tritos, N.A., and Miller, K.K. (2023). Diagnosis and Management of Pituitary Adenomas: A Review. JAMA *329*, 1386–1398. https://doi.org/10.1001/jama.2023.5444.

6. Song, Z.J., Reitman, Z.J., Ma, Z.Y., Chen, J.H., Zhang, Q.L., Shou, X.F., Huang, C.X., Wang, Y.F., Li, S.Q., Mao, Y., et al. (2016). The genome-wide mutational landscape of pituitary adenomas. Cell Res. *26*, 1255–1259. https://doi.org/10.1038/cr.2016.114.

7. Chen, Z., Jia, Q., Zhao, Z., Zhang, Q., Chen, Y., Qiao, N., Ye, Z., Ji, C., Zhang, Y., He, W., et al. (2022). Transcription Factor ASCL1 Acts as a Novel Potential Therapeutic Target for the Treatment of the Cushing's Disease. J. Clin. Endocrinol. Metab. *107*, 2296–2306. https://doi.org/10.1210/clinem/dgac280.

8. Khetchoumian, K., Balsalobre, A., Mayran, A., Christian, H., Chénard, V., St-Pierre, J., and Drouin, J. (2019). Pituitary cell translation and secretory capacities are enhanced cell autonomously by the transcription factor Creb3l2. Nat. Commun. *10*, 3960. https://doi.org/10.1038/s41467-019-11894-3.

9. Zhang, F., Tanasa, B., Merkurjev, D., Lin, C., Song, X., Li, W., Tan, Y., Liu, Z., Zhang, J., Ohgi, K.A., et al. (2015). Enhancer-bound LDB1 regulates a corticotrope promoter-pausing repression program. Proc. Natl. Acad. Sci. USA *112*, 1380–1385. https://doi.org/10.1073/pnas.1424228112.

10. Asa, S.L., Mete, O., Perry, A., and Osamura, R.Y. (2022). Overview of the 2022 WHO Classification of Pituitary Tumors. Endocr. Pathol. *33*, 6–26. https://doi.org/10.1007/s12022-022-09703-7.

11. Liu, J., Lin, C., Gleiberman, A., Ohgi, K.A., Herman, T., Huang, H.P., Tsai, M.J., and Rosenfeld, M.G. (2001). Tbx19, a tissue-selective regulator of POMC gene expression. Proc. Natl. Acad. Sci. USA *98*, 8674–8679. https://doi.org/10.1073/pnas.141234898.

12. Lamolet, B., Pulichino, A.M., Lamonerie, T., Gauthier, Y., Brue, T., Enjalbert, A., and Drouin, J. (2001). A pituitary cell-restricted T box factor, Tpit, activates POMC transcription in cooperation with Pitx homeoproteins. Cell *104*, 849–859. https://doi.org/10.1016/s0092-8674(01)00282-3.

13. Newton, K., Strasser, A., Kayagaki, N., and Dixit, V.M. (2024). Cell death. Cell *187*, 235–256. https://doi.org/10.1016/j.cell.2023.11.044.

14. Hua, P., Liang, R., Yang, S., Tu, Y., and Chen, M. (2024). Microneedle-assisted dual delivery of PUMA gene and celastrol for synergistic therapy of rheumatoid arthritis through restoring synovial homeostasis. Bioact. Mater. *36*, 83–95. https://doi.org/10.1016/j.bioactmat.2024.02.030.

15. Hong, S.S., Marotte, H., Courbon, G., Firestein, G.S., Boulanger, P., and Miossec, P. (2017). PUMA gene delivery to synoviocytes reduces inflammation and degeneration of arthritic joints. Nat. Commun. *8*, 146. https://doi.org/10.1038/s41467-017-00142-1.

16. Le Tissier, P.R., and Mollard, P. (2021). Renewing an old interest: Pituitary folliculostellate cells. J. Neuroendocrinol. *33*, e13053. https://doi.org/10.1111/jne.13053.

17. Aibar, S., González-Blas, C.B., Moerman, T., Huynh-Thu, V.A., Imrichova, H., Hulselmans, G., Rambow, F., Marine, J.C., Geurts, P., Aerts, J., et al. (2017). SCENIC: single-cell regulatory network inference and clustering. Nat. Methods *14*, 1083–1086. https://doi.org/10.1038/nmeth.4463.

18. Millington, G.W.M. (2006). Proopiomelanocortin (POMC): the cutaneous roles of its melanocortin products and receptors. Clin. Exp. Dermatol. *31*, 407–412. https://doi.org/10.1111/j.1365-2230.2006.02128.x.

19. Gelain, F., Luo, Z., and Zhang, S. (2020). Self-Assembling Peptide EAK16 and RADA16 Nanofiber Scaffold Hydrogel. Chem. Rev. *120*, 13434–13460. https://doi.org/10.1021/acs.chemrev.0c00690.

20. Wang, R., Wang, Z., Guo, Y., Li, H., and Chen, Z. (2019). Design of a RADA16-based self-assembling peptide nanofiber scaffold for biomedical applications. J. Biomater. Sci. Polym. Ed. *30*, 713–736. https://doi.org/10.1080/09205063.2019.1605868.

21. Kim, M.W., Gao, W., Lichti, C.F., Gu, X., Dykstra, T., Cao, J., Smirnov, I., Boskovic, P., Kleverov, D., Salvador, A.F.M., et al. (2025). Endogenous self-peptides guard immune privilege of the central nervous system. Nature *637*, 176–183. https://doi.org/10.1038/s41586-024-08279-y.

22. Honegger, J., and Grimm, F. (2018). The experience with transsphenoidal surgery and its importance to outcomes. Pituitary *21*, 545–555. https://doi.org/10.1007/s11102-018-0904-4.

23. Balomenaki, M., Vassiliadi, D.A., and Tsagarakis, S. (2022). Cushing's disease: risk of recurrence following trans-sphenoidal surgery, timing and methods for evaluation. Pituitary *25*, 718–721. https://doi.org/10.1007/s11102-022-01226-y.

24. Agrawal, N., Urwyler, S.A., Mehta, S., Karavitaki, N., and Feelders, R.A. (2024). How to manage Cushing's disease after failed primary pituitary surgery. Eur. J. Endocrinol. *191*, R37–R54. https://doi.org/10.1093/ejendo/lvae110.

25. Jones, P.S., and Swearingen, B. (2022). Pituitary surgery in Cushing's disease: first line treatment and role of reoperation. Pituitary *25*, 713–717. https://doi.org/10.1007/s11102-022-01254-8.

26. Nieman, L.K., Biller, B.M.K., Findling, J.W., Murad, M.H., Newell-Price, J., Savage, M.O., and Tabarin, A.; Endocrine Society (2015). Treatment of Cushing's Syndrome: An Endocrine Society Clinical Practice Guideline. J. Clin. Endocrinol. Metab. *100*, 2807–2831. https://doi.org/10.1210/jc.2015-1818.

27. Karavasili, C., and Fatouros, D.G. (2021). Self-assembling peptides as vectors for local drug delivery and tissue engineering applications. Adv. Drug Deliv. Rev. *174*, 387–405. https://doi.org/10.1016/j.addr.2021.04.024.

28. Mahmoudi, N., Wang, Y., Moriarty, N., Ahmed, N.Y., Dehorter, N., Lisowski, L., Harvey, A.R., Parish, C.L., Williams, R.J., and Nisbet, D.R. (2024). Neuronal Replenishment via Hydrogel-Rationed Delivery of Reprogramming Factors. ACS Nano *18*, 3597–3613. https://doi.org/10.1021/acsnano.3c11337.

29. Westphal, M., Hilt, D.C., Bortey, E., Delavault, P., Olivares, R., Warnke, P.C., Whittle, I.R., Jääskeläinen, J., and Ram, Z. (2003). A phase 3 trial of local

chemotherapy with biodegradable carmustine (BCNU) wafers (Gliadel wafers) in patients with primary malignant glioma. Neuro Oncol. *5*, 79–88. https://doi.org/10.1093/neuonc/5.2.79.

30. Carpenter, A.B., Carpenter, A.M., Aiken, R., and Hanft, S. (2021). Oncolytic virus in gliomas: a review of human clinical investigations. Ann. Oncol. *32*, 968–982. https://doi.org/10.1016/j.annonc.2021.03.197.

31. van Solinge, T.S., Nieland, L., Chiocca, E.A., and Broekman, M.L.D. (2022). Advances in local therapy for glioblastoma - taking the fight to the tumour. Nat. Rev. Neurol. *18*, 221–236. https://doi.org/10.1038/s41582-022-00621-0.

32. Asuzu, D.T., Alvarez, R., Fletcher, P.A., Mandal, D., Johnson, K., Wu, W., Elkahloun, A., Clavijo, P., Allen, C., Maric, D., et al. (2022). Pituitary adenomas evade apoptosis via noxa deregulation in Cushing's disease. Cell Rep. *40*, 111223. https://doi.org/10.1016/j.celrep.2022.111223.

33. Hao, Y., Stuart, T., Kowalski, M.H., Choudhary, S., Hoffman, P., Hartman, A., Srivastava, A., Molla, G., Madad, S., Fernandez-Granda, C., et al. (2024). Dictionary learning for integrative, multimodal and scalable single-cell analysis. Nat. Biotechnol. *42*, 293–304. https://doi.org/10.1038/s41587-023-01767-y.

OMTON, Volume *34*

Supplemental information

Hydrogel-based lineage-specific gene therapy prevents recurrence of corticotroph tumors

Junpeng Wang, Zezheng Fang, Jiahao Wang, Xu Han, Fan Feng, Runlu Zhang, Baoteng Han, Guangpan Sun, Yulin Zhang, and Shilei Ni

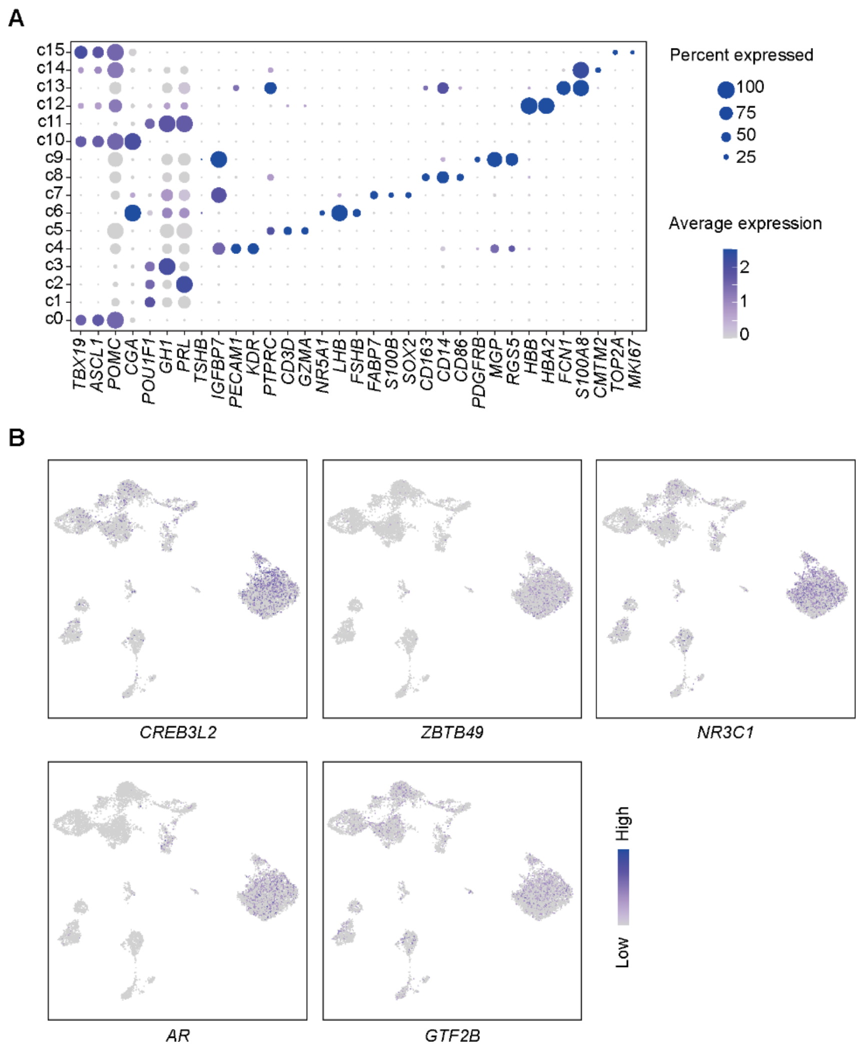

Figure S1. (A) Dot plot depicting marker gene expression across defined clusters. Dot color indicates the average expression level of the gene, and dot size represents the percentage of cells expressing the gene within each cluster. **(B)** UMAP visualization of specific transcription factors of TPIT-lineage cells. Color intensity (gray to blue) reflects increasing expression levels.

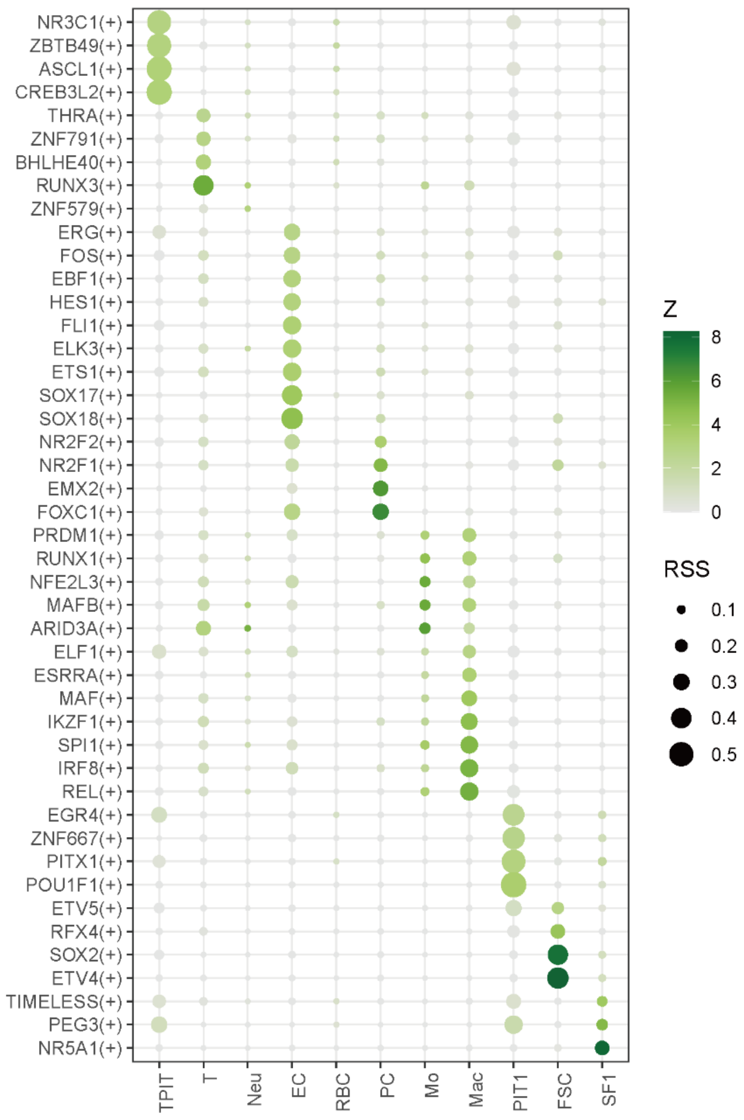

Figure S2. Dot plot showing activation status of cell-type-specific transcription factors (Z-score ≥ 2.5). Color represents Z-score (regulon activity), and dot size indicates RSS (regulon specificity) across clusters. TPIT, TPIT-lineage cells. T, T cells. Neu, neutrophil. EC, endothelial cell. RBC, red blood cells. PC, pericyte. Mo, monocyte. Mac, macrophage. PIT1, PIT1-lineage cells. FSC, folliculostellate cells. SF1, SF1-lineage cells.

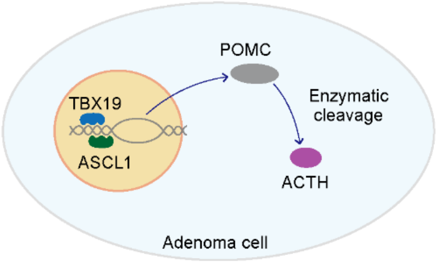

Figure S3. Schematic diagram of ACTH production process in corticotroph tumor cells.

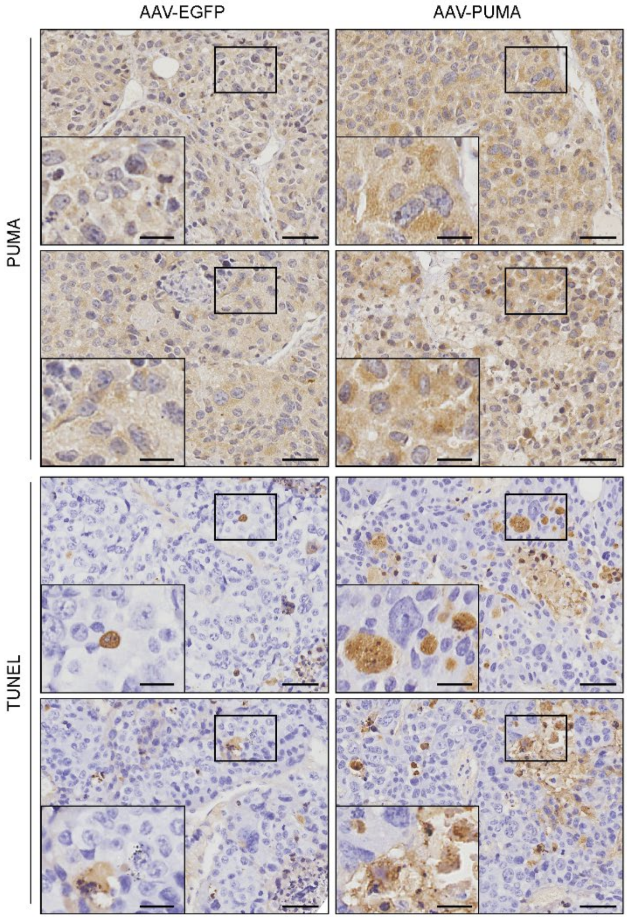

Figure S4. Representative IHC images of PUMA expression and TUNEL staining for apoptosis in tumor tissues. Scale bars: 50 μm (main panels), 20 μm (insets).

A

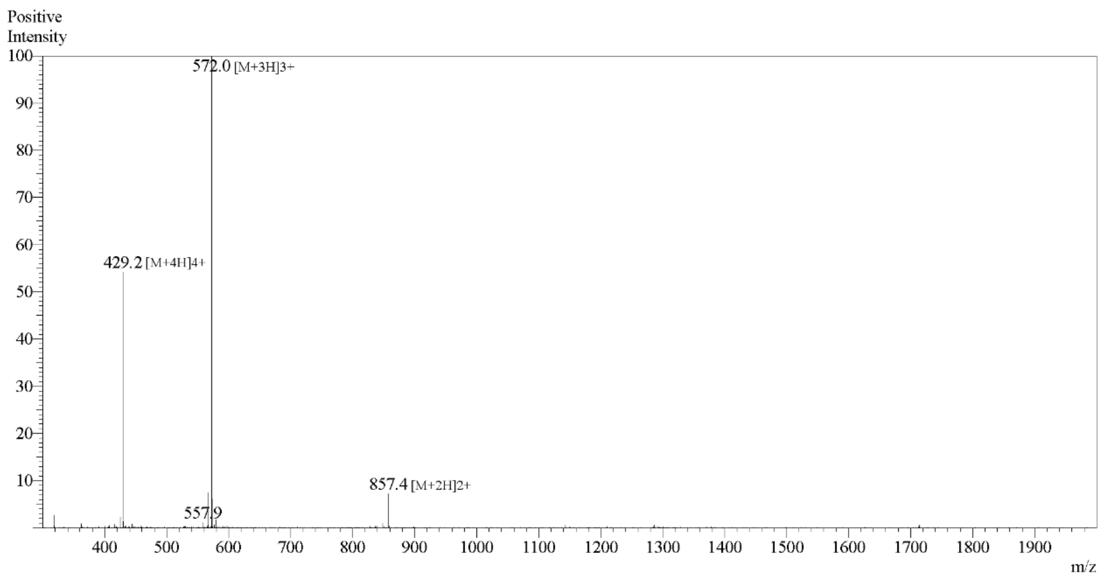

B

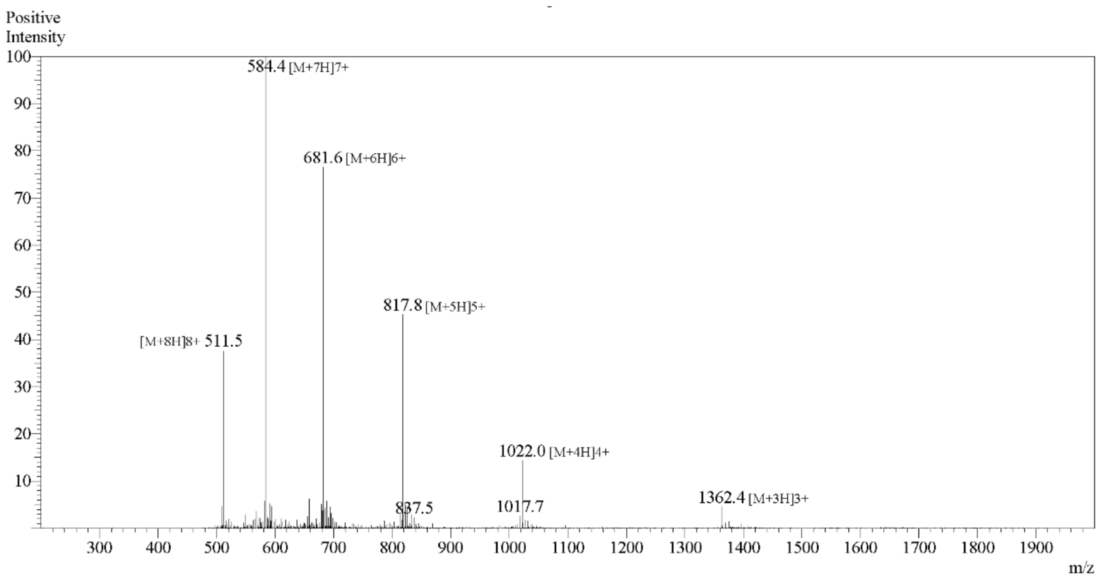

Figure S5. Mass spectrometry profiles of peptides RADA16 (A) and RLM38 (B).

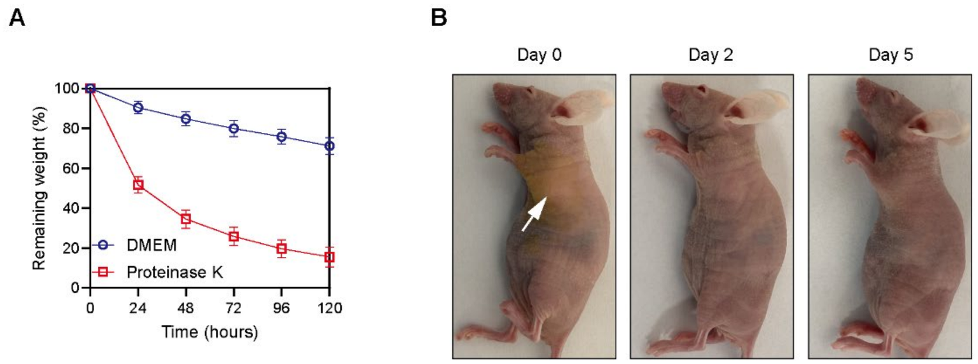

Figure S6. (A) Degradation of the blank hydrogel in DMEM with or without proteinase K. Data represent mean ± SD. n = 3. **(B)** Photograph of a mouse after subcutaneous injection of 30 µL blank hydrogel. The white arrow indicates the wheal formed at the injection site immediately following hydrogel administration.

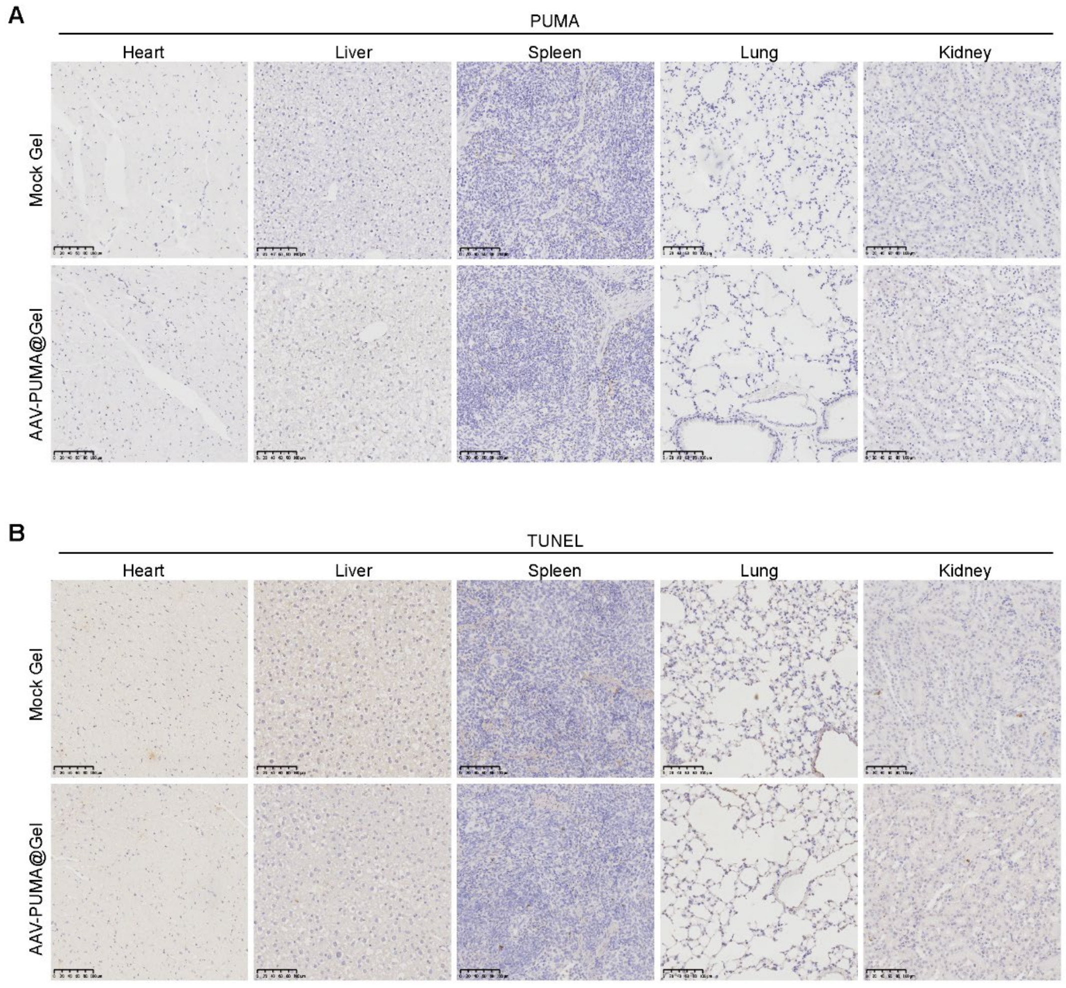

Figure S7. Representative IHC images of PUMA expression **(A)** and TUNEL staining for apoptosis **(B)** in heart, liver, spleen, lung, and kidney tissues. Scale bars: 100 µm.

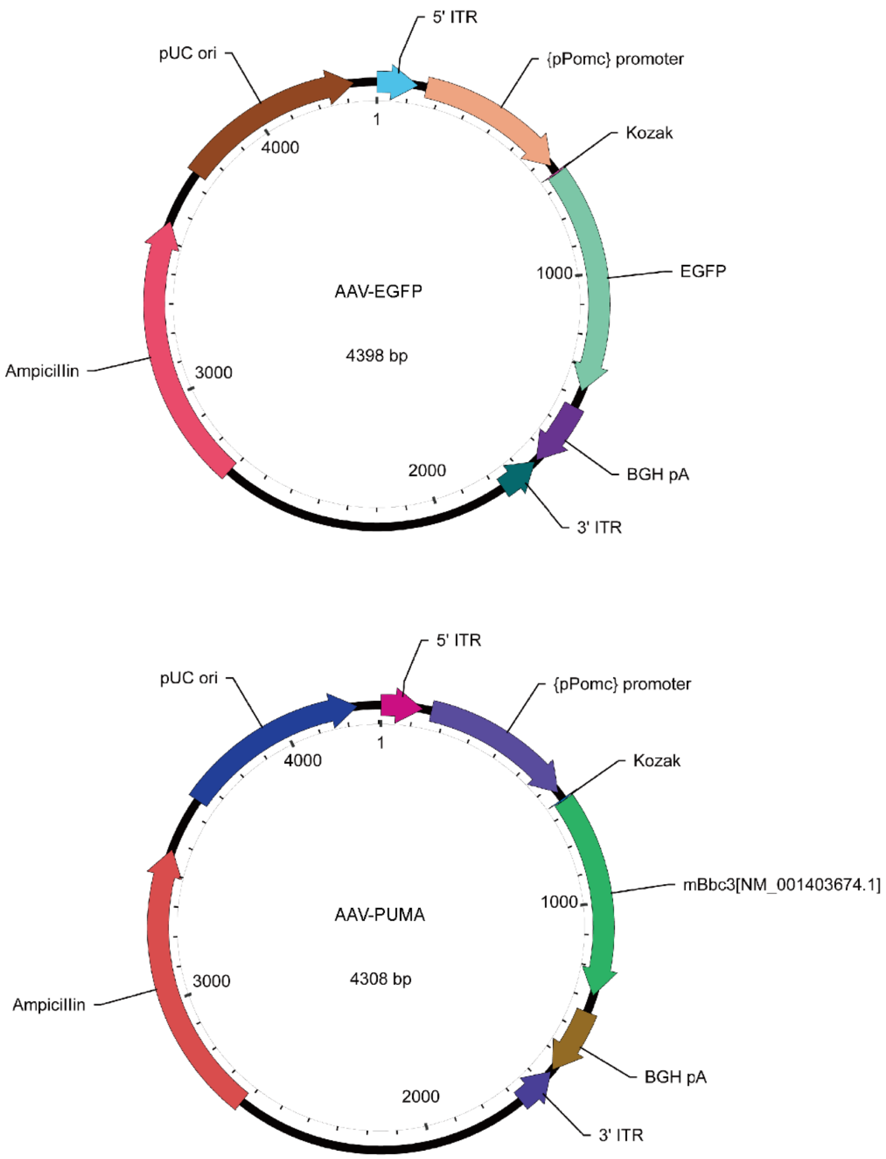

Figure S8. Plasmid maps of AAV-EGFP and AAV-PUMA.

Table S1. Sequences of siRNAs

siRNAs	Type	Sequences
siTBX19-1	sense	5'-GGAGAUAACAGCUCUCAAAdTdT-3'
	antisense	5'-UUUGAGAGCUGUUAUCUCCdTdT-3'
siTBX19-2	sense	5'-GGAUGUUCCCUGUCCUGAAdTdT-3'
	antisense	5'-UUCAGGACAGGGAACAUCCdTdT-3'
siNC	sense	5'-UUCUCCGAACGUGUCACGUdTdT-3'
	antisense	5'-ACGUGACACGUUCGGAGAAdTdT-3'

Table S2. Sequences of primers

Primers	Type	Sequences
Tbx19	forward	5'-GCCTGCTTAACGTGGTGGAA-3'
	reverse	5'-ACATCCGTCTGCCGTTCTTG-3'
Puma	forward	5'-CAGCACTTAGAGTCGCCCG-3'
	reverse	5'-GTGAGGGTCGGTGTCGATG-3'
Pomc	forward	5'-ATGCCGAGATTCTGCTACAGT-3'
	reverse	5'-CCACACATCTATGGAGGTCTGAA-3'
Gapdh	forward	5'-TGTCTCCTGCGACTTCAACA-3'
	reverse	5'- GGTGGTCCAGGGTTTCTTACT-3'
